# Two New Terpenoids from *Talaromyces purpurogenus*

**DOI:** 10.3390/md16050150

**Published:** 2018-05-02

**Authors:** Wenjing Wang, Xiao Wan, Junjun Liu, Jianping Wang, Hucheng Zhu, Chunmei Chen, Yonghui Zhang

**Affiliations:** Hubei Key Laboratory of Natural Medicinal Chemistry and Resource Evaluation, Tongji Medical College, Huazhong University of Science and Technology, Wuhan 430030, China; wangwj0122@163.com (W.W.); marina.wanx@gmail.com (X.W.); junjun.liu@hust.edu.cn (J.L.); jpwang1001@163.com (J.W.); zhuhucheng@hust.edu.cn (H.Z.)

**Keywords:** sesquiterpenoid, diterpenoid, *Talaromyces purpurogenus*, NMR data calculations, ECD calculations, cytotoxicities

## Abstract

A new sesquiterpenoid 9,10-diolhinokiic acid (**1**) and a new diterpenoid roussoellol C (**2**), together with 4 known compounds, were isolated from the extracts of laboratory cultures of marine-derived fungus *Talaromyces purpurogenus*. 9,10-diolhinokiic acid is the first thujopsene-type sesquiterpenoid containing a 9,10-diol moiety, and roussoellol C possesses a novel tetracyclic fusicoccane framework with an unexpected hydroxyl at C-4. These new structures were confirmed by spectroscopic data, chemical method, NMR data calculations and electronic circular dichroism (ECD) calculations. The selected compounds were evaluated for cytotoxicities against five human cancer cell lines, including SW480, HL-60, A549, MCF-7, and SMMC-7721 and the IC_50_ values of compound **2** against MCF-7 and **3** against HL-60 cells were 6.5 and 7.9 μM, respectively.

## 1. Introduction

Over the past forty years, more than 60% small molecule new drugs have been directly or indirectly derived from natural product source, which demonstrates that natural products continue to play a significant role in drug discovery and development process [1]. Fungi-derived natural products are rich sources of medicines due to their diverse chemical structures and bioactivities. For example, lovastatin, penicillin, echinocandin B, and cyclosporine A have been clinically used as effective medicines, illustrating the significance of fungi-derived metabolites in drug discovery [2].

**Figure 1:**
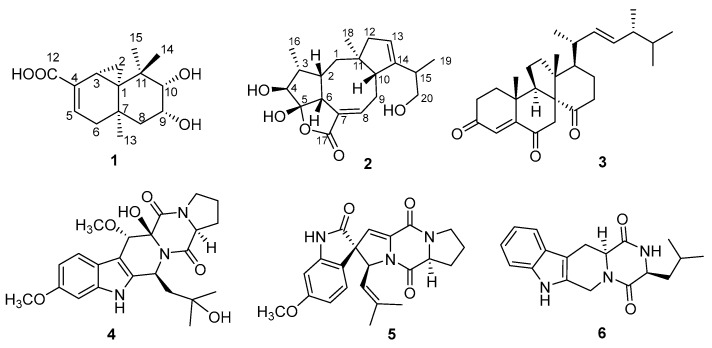
The structures of compounds **1**–**6.**

The fungus *Talaromyces purpurogenus*, previously known as *Penicillium purpurogenum* [3], is widely distributed in terrestrial plants, soil, and marine habitats, and has been reported to produce various secondary metabolites such as meroterpenoids [4,5], polyketides [6,7,8,9,10], lipopeptides [10], and sterols [11]. Meanwhile, the impressive structurally diverse metabolites from this fungus exhibit extensive bioactivities including anti-inflammatory [11], anti-influenza virus [7], insecticidal [4], antitumor [9], and antifungal activities [8]. In our screening of extracts of several fungi for their cytotoxic activities, EtOAc extract of *T. purpurogenus*, isolated from a mud sample, showed significant cytotoxic activity in vitro. A chemical investigation of the fungus *T. purpurogenus* resulted in the isolation of two new secondary metabolites 9,10-diolhinokiic acid (**1**) and roussoellol C (**2**), and four known compounds including dankasterone (**3**) [12,13], cyclotryprostatin E (**4**) [14], 6-methoxyspirotryprostatin B (**5**) [15], and (3*S*,12a*S*)-3-methyl-2,3,6,7,12,12a-hexahydropyrazino[1′,2′:1,6]pyrido[3,4-b]indole-1,4-dion (**6**) (Figure 1) [16]. Details of isolation, structural elucidation, and cytotoxic activities are presented here.

## 2. Results and Discussion

### 2.1. Chemical Identification of Isolated Terpenoids

Compound **1** was isolated as pale yellow oil. The molecular formula of C_15_H_22_O_4_, containing 5 degrees of unsaturation, was deduced from its HRESIMS spectrum (*m*/*z* 265.1450 [M − H]^−^; calcd. for C_15_H_21_O_4_, 265.1440). The IR spectrum gave a hydroxyl absorption band at 3432 cm^−1^ and unsaturated carboxyl and double bond absorbances at 1696 and 1647 cm^−1^, respectively. The ^1^H NMR data (Table 1) of **1** showed the existence of one olefinic proton at *δ*_H_ 6.56 (1H, dd, *J* = 7.0, 1.8 Hz); two oxygenated protons at *δ*_H_ 3.23 (1H, d, *J* = 3.8 Hz) and 4.03 (1H, ddd, *J* = 3.8, 3.7, 3.1 Hz); three methyl signals at *δ*_H_ 1.44, 1.31, and 0.78. The ^13^C NMR and DEPT data (Table 1) showed only 13 carbon resonances, comprising one olefinic carbon [*δ*_C_ 133.7 (C-5)], three methyls [*δ*_C_ 30.6 (C-13), 25.9 (C-14), and 23.5 (C-15)], three methylenes [*δ*_C_ 11.3 (C-2), 42.8 (C-6), and 41.5 (C-8)], three methines including two oxygenated carbons [*δ*_C_ 17.9 (C-3), 72.8 (C-9), and 78.6 (C-10)], as well as three quaternary carbons [*δ*_C_ 35.7 (C-1), 31.9 (C-7), and 39.9 (C-11)]. The missing carbons in the ^13^C NMR including a carboxyl (*δ*_C_ 171.2) and an olefinic carbon (*δ*_C_ 134.3) were revealed by cross-peaks in the HMBC spectrum. These data indicated compound **1** to be a sesquiterpenoid.

**Figure 2:**
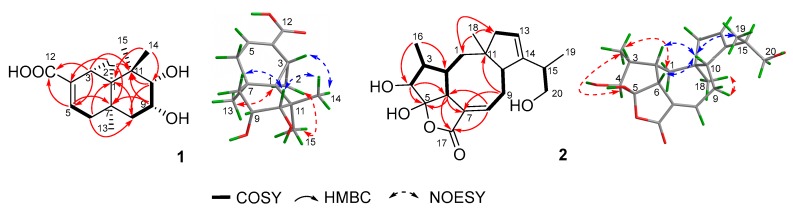
Key 2D correlations of compounds **1** and **2**.

**Figure 3:**
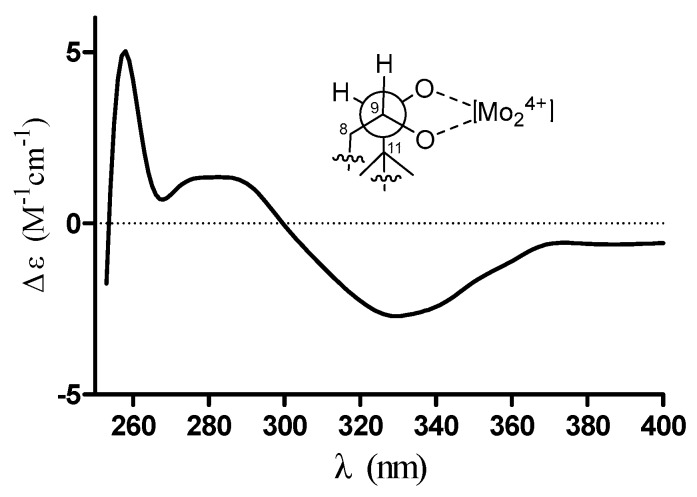
Conformation of the Mo_2_^4+^ complex of compound **1** and its IECD spectrum in DMSO.

Exhaustive analyses of the 2D NMR spectra of **1** revealed some similarity to (+)-thujopsene [17], a sesquiterpenoid derivative isolated from the liverwort *Marchantia polymorpha*. However, two oxygenated methines (*δ*_H_ 4.03, *δ*_C_ 72.8 and *δ*_H_ 3.23, *δ*_C_ 78.6) of **1** replaced the methylenes of (+)-thujopsene and one methyl was oxidized to a carboxyl (*δ*_C_ 171.2) group. The presence of a 9,10-diol moiety was demonstrated by the ^1^H–^1^H COSY cross-peak of H-9 and H-10, and HMBC correlations from H-9 to C-7 and C-11 and from H-10 to C-8 and C-11. The carboxyl was located at C-4, which was substantiated by the HMBC cross-peaks from H-3 and H-5 to C-12 (Figure 2). Therefore, the planar structure of **1** was identified as a 9, 10-diolhinokiic acid [18]. In the NOESY experiment (Figure 2), the correlations of H-2/Me-15 and H-2/Me-13 suggested that these groups were co-facial and assigned as *α*-oriented, while, the interactions of H-3/Me-14 and Me-14/H-10 indicated that they were on the opposite face of the ring system and *β*-oriented. The hydroxyls at C-9 and C-10 were on the same side according to the coupling constant between H-9 and H-10 (*J* = 3.8 Hz). Thus, the relative configuration of **1** was determined. The absolute stereochemistry of the 9,10-diol moiety in **1** was verified by observing the induced electronic circular dichroism (IECD) spectrum after the addition of dimolybdenum tetraacetate in anhydrous DMSO [19,20]. The obvious negative Cotton effect at 310 nm in the IECD spectrum (Figure 3) permitted the 9*R*,10*S* configuration assignment of **1**. Combining with the relative configuration, the absolute stereochemistry of **1** was elucidated as 1*R*,3*R*,7*R*,9*R*,10*S* (Figure 1).

Compound **2** was isolated as colorless oil that gave a [M + Na]^+^ ion peak in the HRESIMS spectrum at *m/z* 371.1839 Δm (calcd. for C_20_H_28_O_5_Na, 371.1834) appropriate for a molecular formula of C_20_H_28_O_5_, corresponding to 7 degrees of unsaturation. The IR spectrum showed a hydroxyl (3430 cm^−1^) and an ester or lactone carbonyl (1742 cm^−1^). The ^1^H NMR data (Table 1) of **2** revealed two olefinic protons at *δ*_H_ 7.04 (1H, m) and 5.31 (1H, s); three oxygenated protons at *δ*_H_ 3.81 (1H, d, *J* = 4.4 Hz), 3.66 (1H, dd, *J* = 10.6, 5.3 Hz), and 3.37 (1H, dd, *J* = 10.6, 7.9 Hz); three methyl signals at *δ*_H_ 0.84 (3H, s), 0.93 (3H, d, *J* = 7.4 Hz), and 1.04 (3H, d, *J* = 6.8 Hz). The ^13^C NMR and DEPT spectra (Table 1) displayed resonances for 20 carbon signals categorized as one carbonyl carbon [*δ*_C_ 173.0 (C-17)], four olefinic carbons [*δ*_C_ 130.5 (C-7), 141.6 (C-8), 122.1 (C-13), and 148.4 (C-14)], one sp^3^ quaternary carbon [*δ*_C_ 46.9 (C-11)], one hemiketal carbon [*δ*_C_ 114.1 (C-1)], six methines including one oxygenated carbon [*δ*_C_ 41.2 (C-2), 40.8 (C-3), 80.0 (C-4), 52.8 (C-6), 47.9 (C-10), and 36.7 (C-15)], four methylenes including one oxygenated carbon [*δ*_C_ 37.6 (C-1), 26.8 (C-9), 45.5 (C-12), and 67.0 (C-20)], and three methyls [*δ*_C_ 11.0 (C-16), 24.9 (C-18), and 17.7 (C-19)]. Consideration of these data and analyses of the ^1^H–^1^H COSY and HMBC spectra (Figure 2) of **2** suggested existence of tetracyclic fusicoccane framework which was similar with that of roussoellol B [21]. Further analyses of the 2D NMR spectra indicated that the methylene at C-4 in roussoellos B was oxygenated (*δ*_C_ 80.0), which was confirmed by ^1^H–^1^H COSY cross-peak of H-3 and H-4 and the HBMC correlations from H-4 to C-3, C-5, C-6, and C-16. In addition, the Δ^10,14^-double bond in roussoello B shifted to C-13 and C-14 which was evidenced by the HBMC correlations from H-13 to C-10, C-11, and C-12. Moreover, HMBC correlations from H-15 and H-19 to the oxygenated methylene carbon at *δ*_C_ 67.0 (C-20) indicated the presence of a hydroxymethyl functionality. Hence, the planar structure of **2** was established as shown. 

**Figure 4:**
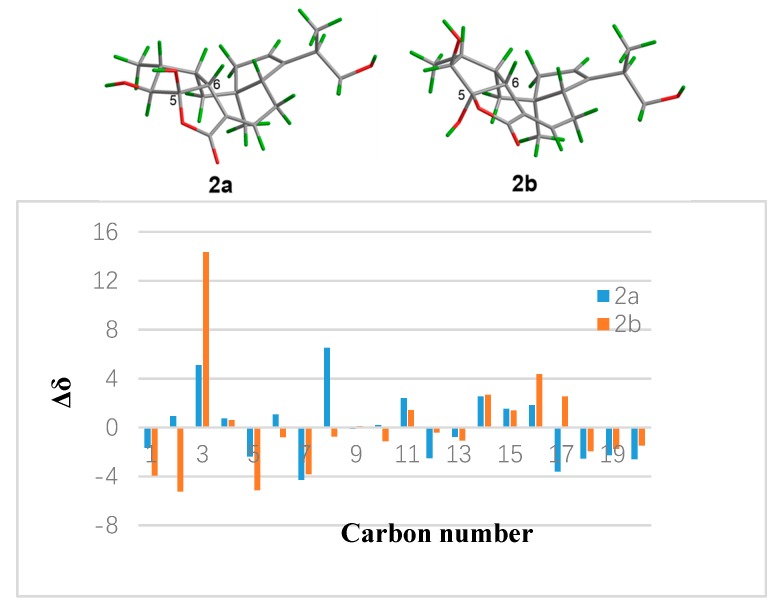
Structures and differences in ppm between calculated and experimental ^13^C NMR shifts for **2a** and **2b**.

In the NOESY experiment (Figure 2), the NOESY correlations of H-2/H-10, H-6/H-10, and H-10/Me-19 indicated the *β*-orientation for these protons. Meanwhile, correlations of H-1/Me-16, Me-16/H-4, and Me-18/H-9 suggested that H-4, Me-16, and Me-18 were on the opposite side and *α*-oriented. Even though the NOESY correlation of H-10/Me-19 were observed, but the configuration of C-15 could not be determined by the NOESY experiment due to the freely rotation of the bond between C-14 and C-15. In order to determine the relative configuration of 5-OH, the theoretical calculation of ^13^C NMR chemical shifts of epimers **2a** and **2b** (Figure 4) were performed to semiempirical PM3 quantum mechanical geometry optimizations using Gaussian09 at the B3LYP/6-31G* level [22]. The experimental shifts were plotted against the calculated shifts, and least-squares fit lines was confirmed. The calculated shifts for **2a** and **2b** were corrected by the slope and intercept to get the corrected ^13^C shifts (Table 1), and the differences between the corrected and experimental ^13^C NMR chemical shifts were analyzed [23,24]. The result showed that the correlation coefficient *R*^2^ of **2a** (0.9966) was higher than that of **2b** (0.9926) (Figures S21 and S22). Meanwhile, the MAE (mean absolute error) and MD (maximum deviation) of **2a** (MAE = 2.28, MD = 6.5) were obviously lower than that of **2b** (MAE = 2.74, MD = 14.3), suggesting that **2a** was more consistent with the experimental values (Figure 4). What’s more, all of the reported fusicoccanes or ophiobolins with 5/8/5/5 ring system possess a *cis*-fused A/D ring [21,25,26,27,28], and the 5-OH of **2** was finally assigned a *β*-orientation as **2a**. 

**Figure 5:**
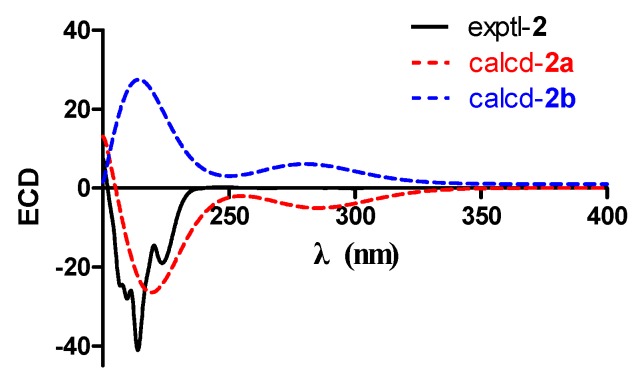
Experimental and calculated ECD spectra of **2**.

To determine the absolute configuration of compound **2**, the electronic circular dichroism (ECD) calculation was performed. The experimental and simulated spectra generated by BALLOON [29,30] were performed to semiempirical PM3 quantum mechanical geometry optimizations using the Gaussian 09 program (Figures S2 and S3, Supplementary Materials) [31]. The ECD spectrum of each conformer was calculated using the TDDFT methodology at B3LYP/6-311++G(d,p)//B3LYP/6-31G(d) level. Comparison of the experimental and calculated spectra of **2** showed more agreement (Figure 5) for the **2a** configuration. The experimental ECD is consistent with the calculated ECD of **2** (Figure 5), indicating a (2*S*,3*R*,4*S*,5*S*,6*R*,10*R*,11*S*)-configuration. Therefore, the structure of **2**, namely, roussoellol C, was deduced as shown.

### 2.2. Cytotoxic Activities of Selected Compounds

The growth inhibitory effects of the selected compounds (**1**–**3**) against human colonic carcinoma cell line (SW480), human promyelocytic leukemia cells (HL-60), human non-small-cell lung cancer cells (A549), breast adenocarcinoma cell line (MCF-7), and human hepatocellular carcinoma cell line (SMMC-7721) were assayed by using MTT method [32], with adriamycin as the positive control. Compounds **1**–**3** exhibited moderate antiproliferative activities against these cells with IC_50_ values ranging from 6.5 to 35.7 μM (Table 2). Normally, cytotoxic natural products display better activities against HL-60 than any other cancer cell lines because HL-60 cells are much sensitive in the assay. However, it is interesting that compound **2** showed significant selectivity toward MCF-7 cells with an IC_50_ value of 6.5 μM but with an IC_50_ value of 10.9 μM against HL-60. Although an IC_50_ value of 6.5 μM does not indicate strong potency, the selectivity of **2** against MCF-7 still makes it a promising lead compound for further studies.

## 3. Materials and Methods 

### 3.1. General Experimental Procedures

Optical rotations were measured on a Rudolph Autopol IV automatic polarimeter with a 0.7 mL cell (Rudolph Research Analytical, Hackettstown, NJ, USA). UV spectra were recorded with a PerkinElmer Lambda 35 spectrophotometer (PerkinElmer, Inc., Fremont, CA, USA). IR spectra were measured on a Bruker Vertex 70 FT-IR spectrophotometer (Bruker, Karlsruhe, Germany). ECD data were obtained with a JASCO-810 instrument (JASCO Co., Ltd., Tokyo, Japan). HRESIMS data were recorded on a Thermo Fisher LTQ XL LC/MS (Thermo Fisher, Palo Alto, CA, USA). 1D and 2D NMR spectra were measured with a Bruker AM-400 NMR spectrometer at 25 °C (Bruker, Karlsruhe, Germany), the NOESY mixing time was 100 ms. Compounds were purified by an Agilent 1220 HPLC (Agilent Technologies Inc., Santa Clara, CA, USA) system semi-preparative HPLC equipped with a UV detector (Agilent Technologies Inc.). Column chromatography was performed on silica gel (100–200 and 200–300 mesh, Qingdao Marine Chemical Inc., Qingdao, China), Sephadex LH-20 (Pharmacia Biotech AB, Uppsala, Sweden) and ODS (50 μm, YMC, Kyoto, Japan).

### 3.2. Fungal Material

The fungus PP-414 was isolated from a mud sample collected on the coastal beach in Qinghuangdao County, Hebei Province, China. The mud sample (5 g) was suspended in 50 mL sterile water with a concentration at 10^–1^ g/mL and then every 0.5 mL mutterlauge was respectively diluted to 10^–2^, 10^–3^, 10^–4^ g/mL with sterile water. Each sample was coated individually on potato dextrose agar (PDA) medium contained chloramphenicol, and incubated at 28 °C to get single colonies by routine microbiological methods. The internal transcribed spacer (ITS) region was amplified by PCR using primers ITS1 (5′-TCCGTAGGTGAACCTGCGG-3′) and ITS4 (5′-TCCTCCGCTTATTGATATGC-3′), then submitted to GenBank and identified as *Talaromyces purpurogenus* by ITS sequence homology (99% similarity with *Talaromyces purpurogenus* strain Q2, accession no. KX432212.1 (max score 974, query cover 96%, *e* value 0.0)) and physiological characteristics with accession no. MH120320. The voucher sample, PP-414, has been preserved in the culture collection center of Tongji Medical College, Huazhong University of Science and Technology (Wuhan, China).

### 3.3. Fermentation and Isolation

The fungus PP-414 was incubated on potato dextrose agar (PDA) at 28 °C for 7 days, the agar cultures were cut into small pieces (approximately 0.5 × 0.5 × 0.5 cm^3^) and then inoculated into 100 × 1 L Erlenmeyer flasks which containing 250 g rice and 250 mL distilled water. After incubating at 28 °C for 28 days, the solid rice medium was distilled with CH_3_CH_2_OH and then extracted three times with EtOAc. The EtOAc extract (80 g) was chromatographed on silica gel chromatography column (CC, 80–120 mesh) eluting with petroleum ether/EtOAc (100:0–0:1, *v*/*v*) to afford five fractions (Fr. A-Fr. E). Fr. C (4.5 g) was further separated by Sephadex LH-20 (CH_2_Cl_2_/MeOH 1:1) and silica gel CC (200–300 mesh) eluting with CH_2_Cl_2_/MeOH (200:1–20:1, *v*/*v*) to obtain four fractions (C1-C5), Fr. C2 was further purified by semi-preparative HPLC (MeCN-H_2_O, 85:15, *v*/*v*) to yield **3** (9.0 mg, *t*_R_ = 52.5 min). Fr. C3 was further purified by semi-preparative HPLC (MeCN-H_2_O, 45:55, *v*/*v*) to obtain **6** (6.0 mg, *t*_R_ = 35.2 min). Fr. C4 was further separated by Sephadex LH-20 (MeOH) and semi-preparative HPLC (MeOH-H_2_O, 65:35, *v*/*v*) to yield **4** (4.5 mg, *t*_R_ = 20.0 min) and **5** (5.0 mg, *t*_R_ = 46.0 min).

Fr. D (8.5 g) was further separated by reversed-phase MPLC (MeOH/H_2_O, 10:90–100:0) to obtain seven fractions (D1–D7), Fr. D6 was purified by Sephadex LH-20 (CH_2_Cl_2_/MeOH 1:1) and silica gel CC (200–300 mesh) eluting with CH_2_Cl_2_/MeOH (200:1–10:1, *v*/*v*) to obtain four fractions (D6.1–D6.4), Fr. D6.3 was further purified by semi-preparative HPLC (MeOH-H_2_O, 65:35, *v*/*v*) to yield **1** (2.5 mg, *t*_R_ = 16.0 min). Fr. D6.4 was further separated by semi-preparative HPLC (MeOH-H_2_O, 55:45, *v*/*v*) to yield **2** (1.1 mg, *t*_R_ = 27.5 min). 

**Compound 1**: pale yellow oil (MeOH), [*α*]${}_{\text{D}}^{25}$ + 43.0 (*c* 0.1, MeOH); UV (MeOH) *λ*_max_ (log *ε*) 203 (3.71), 235 (3.51) nm; CD (MeOH) *λ*_max_ (Δ*ε*) 203 (−1.37), 233 (2.17), 263 (0.54), 282 (0.72), 335 (−0.13) nm; IR *ν*_max_ 3432, 2923, 1696, 1647, 1382, 1246, 1062 cm^–1^; ^1^H and ^13^C NMR data, see Table 1; HRESIMS *m/z* 265.1450 [M − H]^−^ (calcd. for C_15_H_21_O_4_, 265.1440).

**Compound 2**: colorless oil (MeOH), [*α*]${}_{\text{D}}^{25}$ − 4.0 (*c* 0.05, MeOH); UV (MeOH) *λ*_max_ (log *ε*) 202 (3.87), 212 (3.81), 225 (3.84) nm; CD (MeOH) *λ*_max_ (Δ*ε*) 212 (−7.28), 232 (−2.44), 243 (−3.34) nm; IR *ν*_max_ 3430, 2928, 1742, 1631, 1384, 1218, 1027 cm^–1^; ^1^H and ^13^C NMR data, see Table 1; HRESIMS *m/z* 371.1839 [M + Na]^+^ (calcd. for C_20_H_28_O_5_Na, 371.1834).

### 3.4. NMR Calculation

The ^13^C NMR chemical shifts of each conformer were calculated at the B3LYP/6-311++G(d,p)//B3LYP/6-31G(d) level by the IEFPCM solvation model implemented using Gaussian 09 program with MeOH as solvent, which were then combined using Boltzmann weighting according to their population contributions. The detailed methods were the same as previously described [33].

### 3.5. ECD Calculation

The electronic circular dichroism (ECD) spectra of each conformer were calculated by the TDDFT methodology with MeOH as solvent. The detailed methods were the same as previously described [32] The ECD spectra of each conformer were simulated using a Gaussian function with a bandwidth σ of 0.4 eV. The spectra were combined after Boltzmann weighting according to their population contributions and UV correction was applied.

### 3.6. Cytotoxicity against Cancer Cell Lines

Cytotoxicity of the selected compounds against the five cancer cell lines (SW480, HL-60, A549, MCF-7, and SMMC-7721) was evaluated by the MTT method with adriamycin as positive control. All cells were cultured in RPMI.1640 medium contained 10% fetal bovine serum, 2 mM l-glutamine, 100 U/mL penicillin, and 100 μg/mL streptomycin at 37 °C in a humidified atmosphere with 5% CO_2_. Tumor cells were seeded in 96-well microtiter plates at 5000 cells/wel, and the test compounds at concentrations ranging from 1.56 to 50 µM were added to the wells 12 h later. After incubation for 48 h, the metabolic conversion of 20 μL of MTT (5 mg/mL) 3-(4,5-dimethylthiazol-2-yl)-2,5-diphenyltetrazolium bromide was added and the incubation was continued for 4 h at 37 °C. The medium was exchanged with the medium containing 100 μL triplex solution of 10% SDS, 5% isopropyl alcohol and 12 mM HCl and then cultured 12–20 h at 37 °C. The results were obtained using a microplate spectrophotometer plate reader at 570 nm and the value of inhibition was calculated by formula: % inhibition = [(OD_control_ − OD_treated_)/OD_control_] × 100%. Selected compounds were tested at five concentrations (50, 25, 12.5, 6.25, 3.12 and 1.56 μM) in 100% DMSO with a final concentration of DMSO was 0.5% (*v*/*v*) in each well. The IC_50_ values were calculated by the means ± SEM calculating by GraphPad Prism 5.

## 4. Conclusions

In conclusion, we have reported six metabolites, including two new structures from the culture extract of *T. purpurogenus*. Among them, 9,10-diolhinokiic acid (**1**) is the first reported thujopsene-type sesquiterpenoid containing a 9,10-diol moiety and roussoellol C (**2**) possesses a novel tetracyclic fusicoccane diterpenoid with an unexpected hydroxyl at C-4. This study further enriched the structure diversity of secondary metabolites of this species. Additionally, compound **2** showed significant selectivity aginst MF-7 with IC_50_ values of 6.5 μM, which makes it a promising lead compound for further studies.

## Figures and Tables

**Table 1:** ^1^H (400 MHz) and ^13^C NMR (100 MHz) Data for Compound **1** and **2** (CD_3_OD) and DFT Calculation of ^13^C NMR for **2a** and **2b.**

Position	1		2		2a		2b	
	*δ*_H_ (*J* in Hz)	*δ*_C_	*δ*_H_ (*J* in Hz)	*δ*_C_	*δ*_C_ (calcd.)	*δ*_C_ (cor)	*δ*_C_ (calcd.)	*δ*_C_ (cor)
1	-	35.7	1.60, d, 13.0 1.40, dd, 14.6, 13.0	37.6	39.2	35.9	36.2	33.7
2	0.85, dd, 9.1,5.0	11.3	2.70, m	41.2	45.7	42.1	38.6	36.0
	0.76, d, 5.1							
3	2.11, dd, 9.0, 5.1	17.9	2.45, m	40.8	49.6	45.9	58.7	55.1
4	-	134.3	3.81, d, 4.4	80.0	86.0	80.7	85.4	80.6
5	6.56, d, 4.6	133.7	-	114.1	118.4	111.7	115.2	109.0
6	1.88, dd, 18.3, 2.6	42.8	3.42, br d, 9.4	52.8	57.9	53.8	55.4	52.0
	1.78, dd, 18.3, 7.0							
7	-	31.9	-	130.5	133.5	126.2	133.7	126.7
8	1.57, dd, 14.4, 3.1	41.5	7.04, m	141.6	156.4	148.1	148.6	140.9
	1.50, dd, 14.4, 3.7							
9	4.03, ddd, 3.8, 3.7, 3.1	72.8	2.65, overlap 2.36, m	28.6	31.4	28.5	31.0	28.7
10	3.23, d, 3.8	78.6	3.19, dd, 13.6, 2.8	47.9	51.9	48.1	50.0	46.8
11	-	39.9	-	46.9	53.2	49.3	51.6	48.3
12	-	171.2	2.28, dd, 15.1, 4.2 1.76, dd, 15.1, 2.6	45.5	46.6	43.0	48.2	45.9
13	1.44, s	30.6	5.31, br s	122.1	128.4	121.3	127.8	121.0
14	0.78, s	25.9	-	148.4	159.3	150.9	159.3	151.1
15	1.31, s	23.5	2.22, ddq, 7.9, 5.3, 7.4	36.7	41.6	38.2	40.8	38.1
16			0.93, d, 7.4	11.0	15.0	12.8	17.0	15.4
17			-	173.0	178.6	169.4	184.9	175.5
18			0.84, s	24.9	25.0	22.4	25.0	23.0
19			1.04, d, 6.8	17.7	17.7	15.4	17.5	15.8
20			3.66, dd, 10.6, 5.3 3.37, dd, 10.6, 7.9	67.0	68.9	64.4	69.6	65.5

**Table 2:** Cytotoxicities against Tumor Cells for **1**–**3** (IC_50_, μM).

	1	2	3	Adriamycin
SW480	>40	23.6	14.2	1.2
HL-60	12.6	10.9	7.9	0.05
A549	35.7	25.8	21.3	0.10
MCF-7	>40	6.5	23.8	0.80
SMMC-7721	>40	>40	>40	0.2

## Supplementary Materials

The following are available online at http://www.mdpi.com/1660-3397/16/5/150/s1, HR-ESI-MS, IR, UV and NMR spectra of new compounds **1** and **2**, as well as computational data for compound **2**.
