# Supplementary material for: Two New Terpenoids from Talaromyces purpurogenus
Source: Mar Drugs. 2018 May 2;16(5):150. doi: 10.3390/md16050150 (PMC5983281; doi:10.3390/md16050150)
Supplement: Supplementary file 1 [file marinedrugs-16-00150-s001.pdf]

Two New Terpenoids from *Talaromyces purpurogenus*

Wenjing Wang, Xiao Wan, Junjun Liu, Jianping Wang, Hucheng Zhu, Chunmei Chen * and Yonghui Zhang *

Hubei Key Laboratory of Natural Medicinal Chemistry and Resource Evaluation, Tongji Medical College, Huazhong University of Science and Technology, Wuhan 430030, China; wangwj0122@163.com (W. W.); marina.wanx@gmail.com (X. W.); junjun.liu@hust.edu.cn (J. L.); jpwang1001@163.com (J. W.); zhuhucheng@hust.edu.cn (H. Z.)

* Correspondence: chenchunmei@hust.edu.cn (C. C.); zhangyh@mails.tjmu.edu.cn (Y. Z.); Tel.:+86-27-8369-2892 (C.C.); +86-27-8369-2892 (Y.Z.)

Content

Figure S1. (+)-HR-ESI-MS Spectrum of **1** 3

Figure S2. IR Spectrum of **1** 3

Figure S3. UV Spectrum of **1** 4

Figure S4. ^{1}H NMR Spectrum of **1** in CD_3OD 4

Figure S5. ^{13}C NMR Spectrum of **1** in CD_3OD 5

Figure S6. HSQC Spectrum of **1** in CD_3OD 5

Figure S7. HMBC Spectrum of **1** in CD_3OD 6

Figure S8. ^{1}H–^{1}H COSY Spectrum of **1** in CD_3OD 6

Figure S9. NOESY Spectrum of **1** in CD_3OD 7

Figure S10. (+)-HR-ESI-MS Spectrum of **2** 7

Figure S11. IR Spectrum of **2** 8

Figure S12. UV Spectrum of **2** 8

Figure S13. ^{1}H NMR Spectrum of **2** in CD_3OD 9

Figure S14. ^{13}C NMR Spectrum of **2** in CD_3OD 9

Figure S15. HSQC Spectrum of **2** in CD_3OD 10

Figure S16. HMBC Spectrum of **2** in CD_3OD 10

Figure S17. ^{1}H–^{1}H COSY Spectrum of **2** in CD_3OD 11

Figure S18. NOESY Spectrum of **2** in CD_3OD 11

NMR calculations 11

Figure S19. Optimized geometries of predominant conformers of **2a** at the B3LYP/6-31G(d,p) level. 12

Table S1. Conformational distribution of **2a** 12

Table S2. Compound **2a** structure optimized at B3LYP/6-31G* 13

Figure S20. Optimized geometries of predominant conformers of **2b** at the B3LYP/6-31G(d,p) level 15

Table S3. Conformational distribution of **2b** 15

Table S4. Compound **2b** structure optimized at B3LYP/6-31G* 15

Table S5. DFT Calculation Result for C shifts of **2a** 16

Figure S21. The ^{13}C NMR correlation of experimental data and calculated dada of **2a** 17

Table S6. DFT Calculation Result for C shifts of **2b** 17

Figure S22. The ^{13}C NMR correlation of experimental data and calculated dada of **2b** 18

ECD calculations 18

Table S7. Details for ECD calculation of **2** 18

Figure S1. (+)-HR-ESI-MS Spectrum of **1**

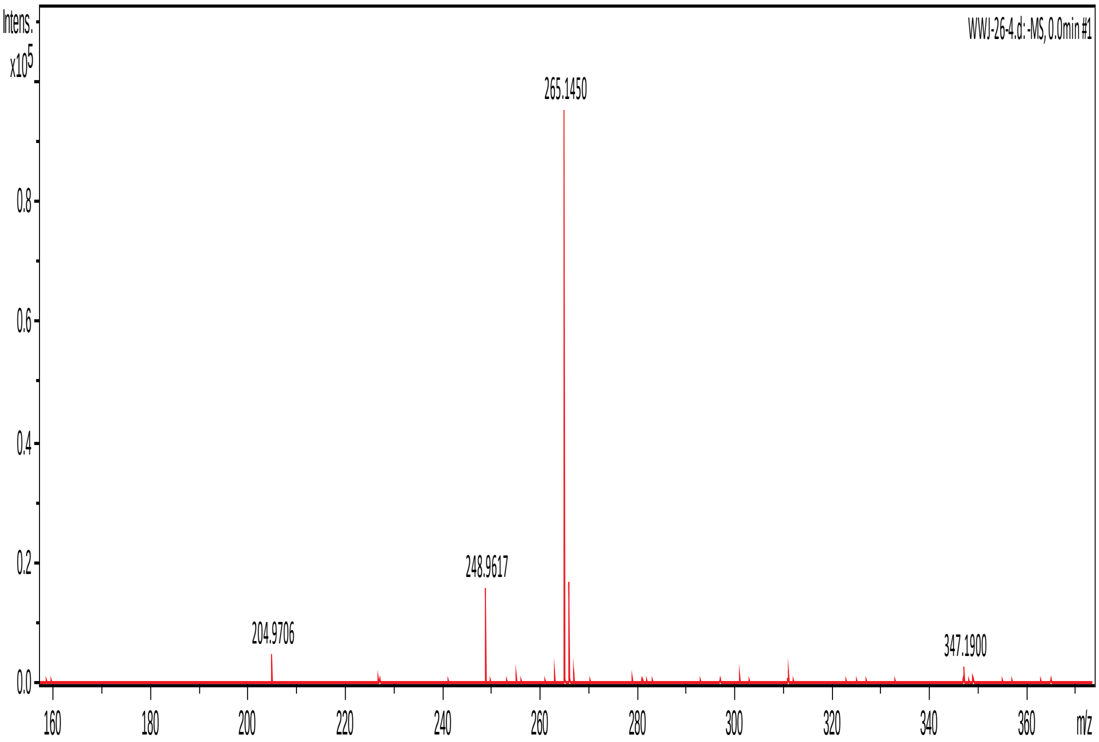

Figure S2. IR Spectrum of **1**

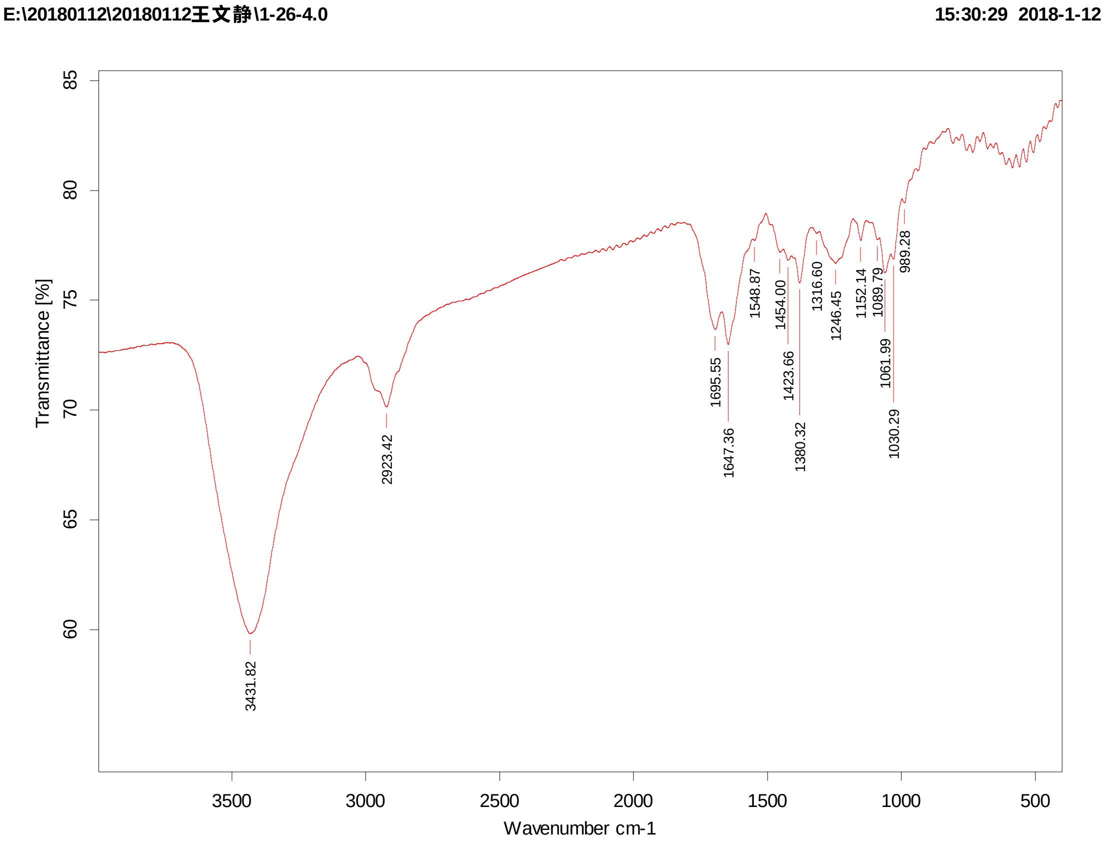

Figure S3. UV Spectrum of **1**

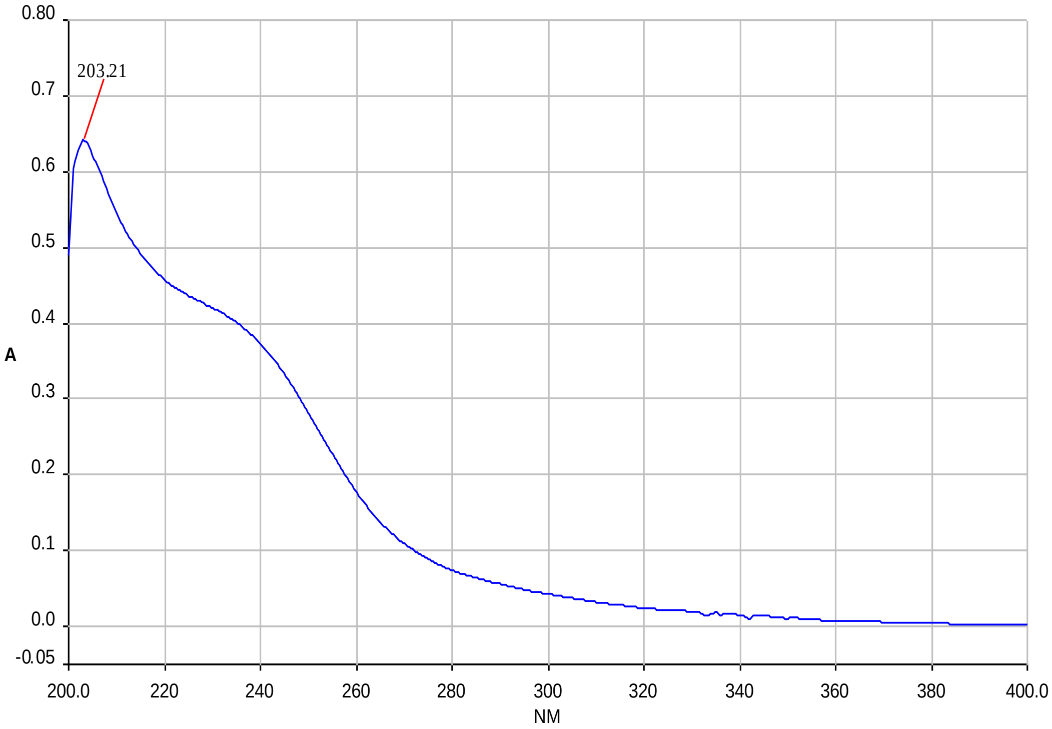

Figure S4. ^{1}H NMR Spectrum of **1** in CD_3OD

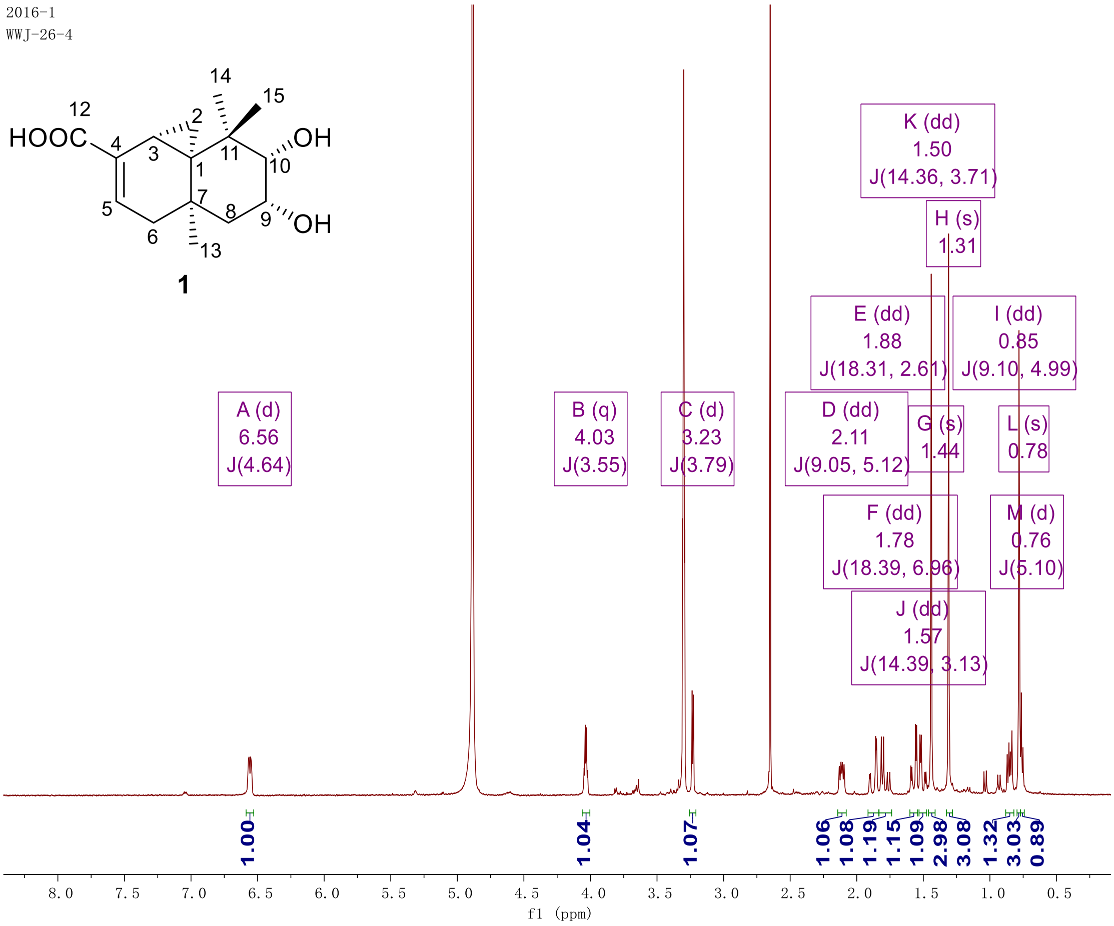

Figure S5. ^{13}C NMR Spectrum of **1** in CD_3OD

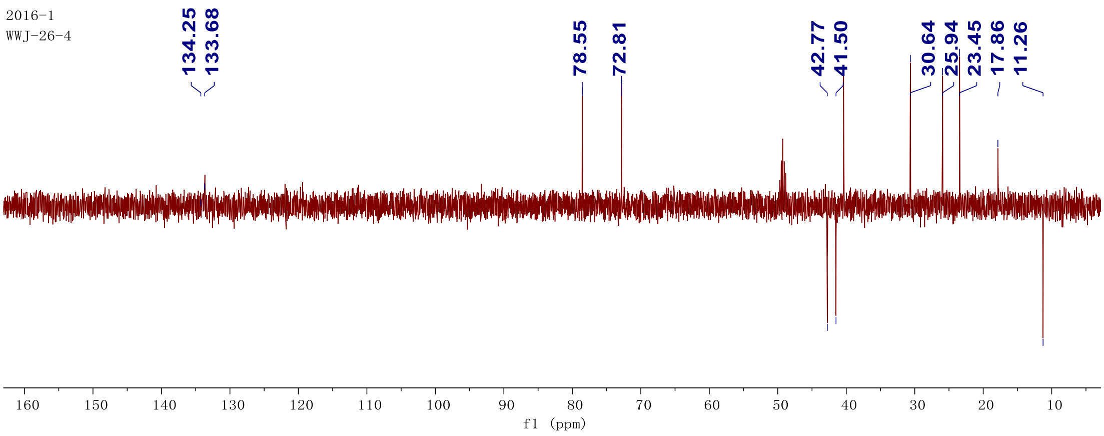

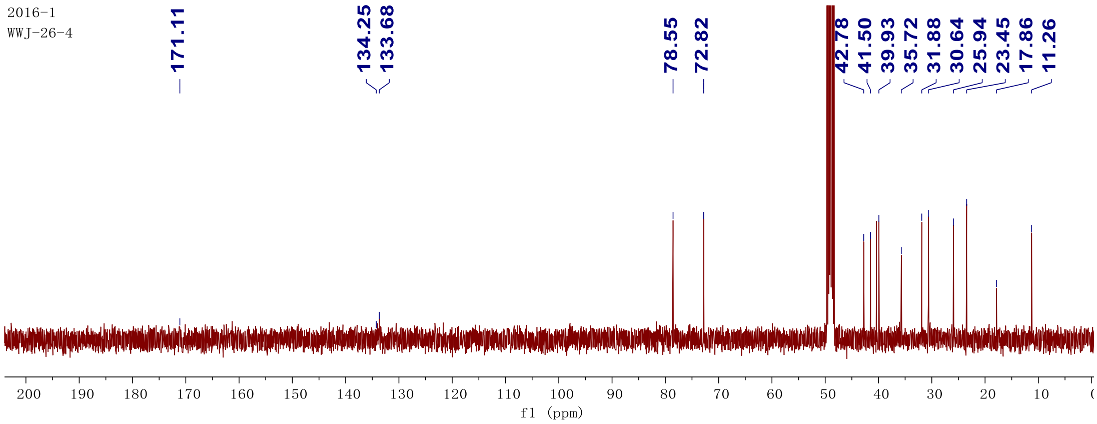

Figure S6. HSQC Spectrum of **1** in CD_3OD

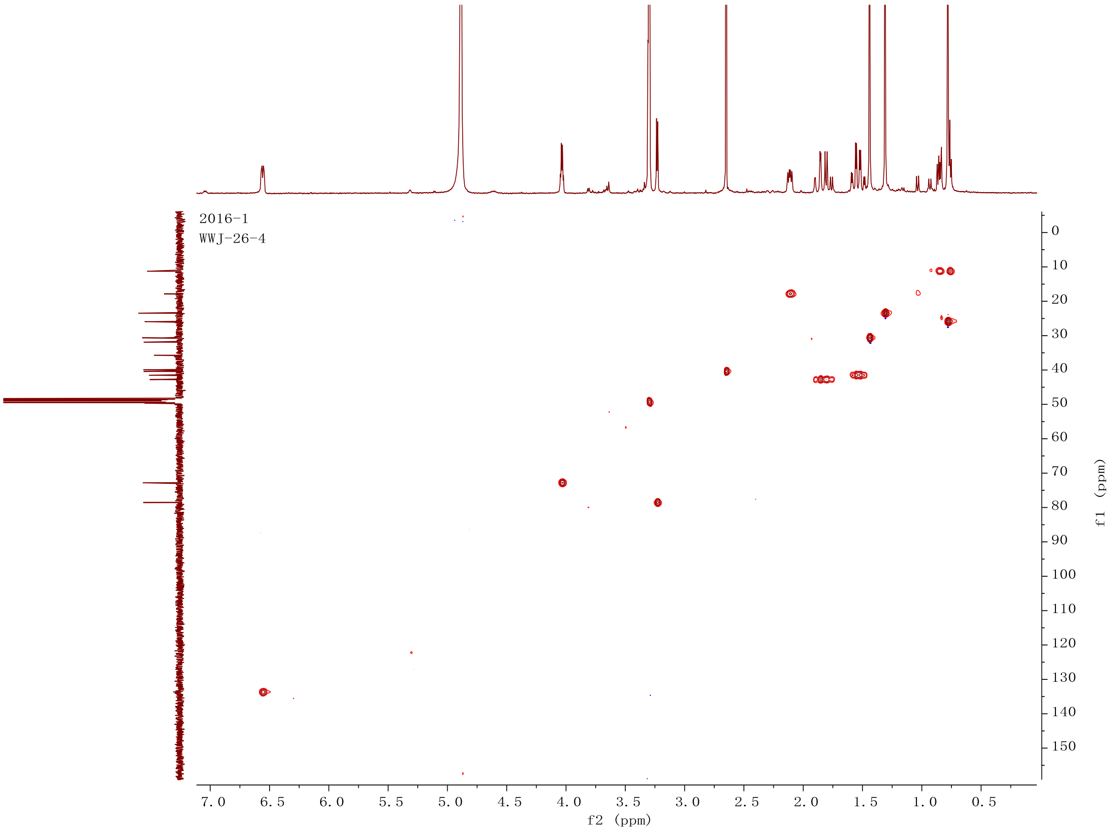

Figure S7. HMBC Spectrum of **1** in CD_3OD

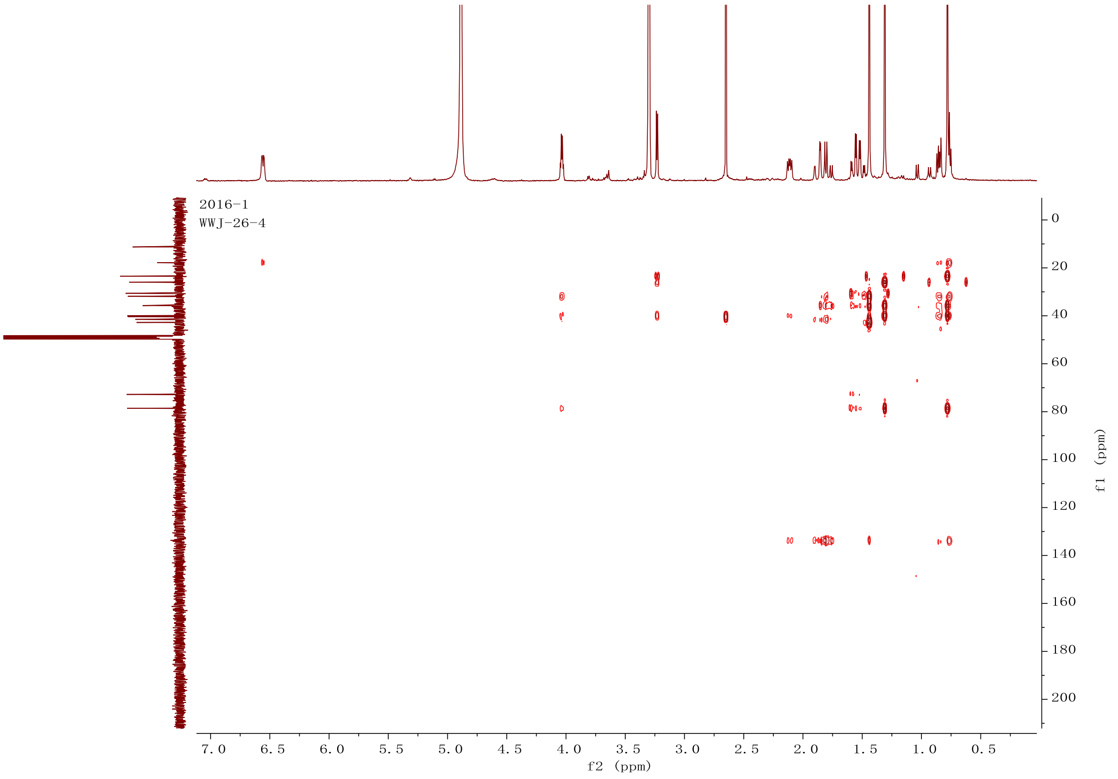

Figure S8. $^1H-^1H$ COSY Spectrum of **1** in CD_3OD

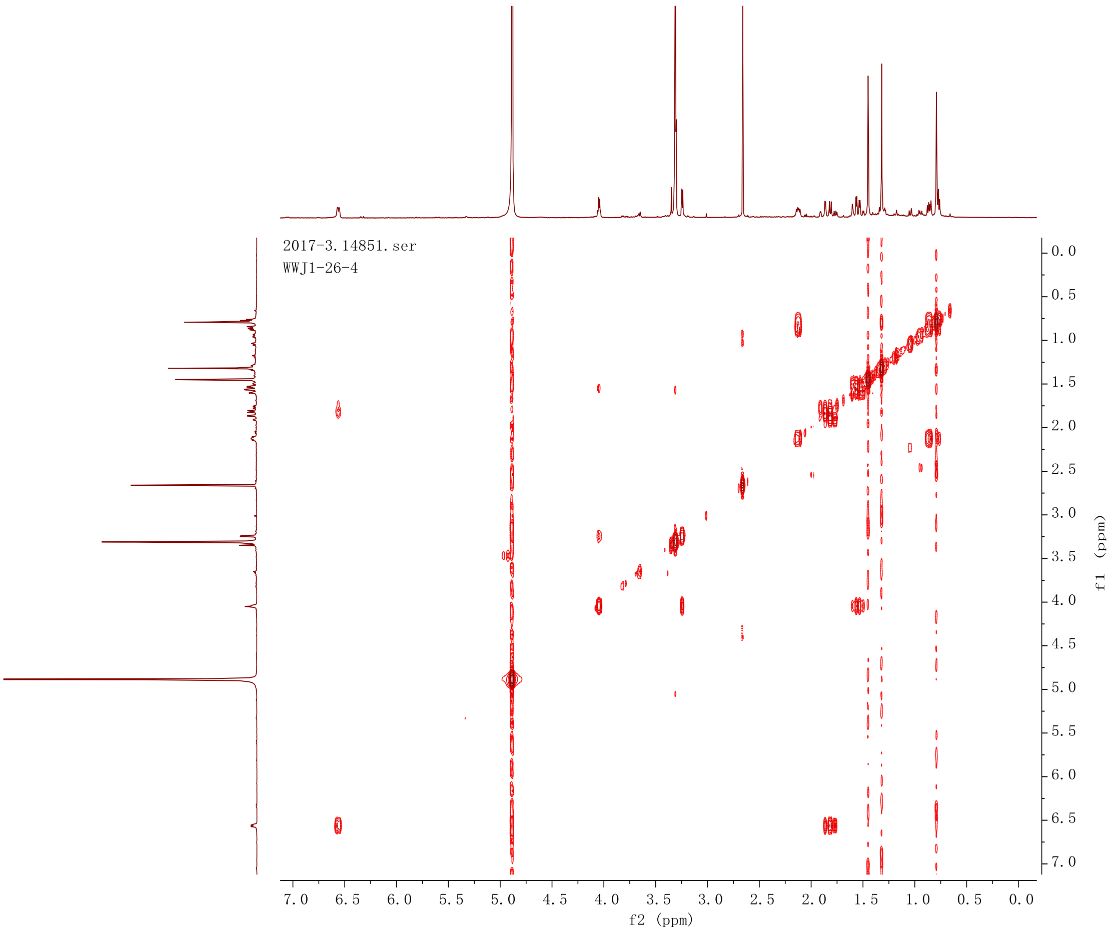

Figure S9. NOESY Spectrum of **1** in CD_3OD

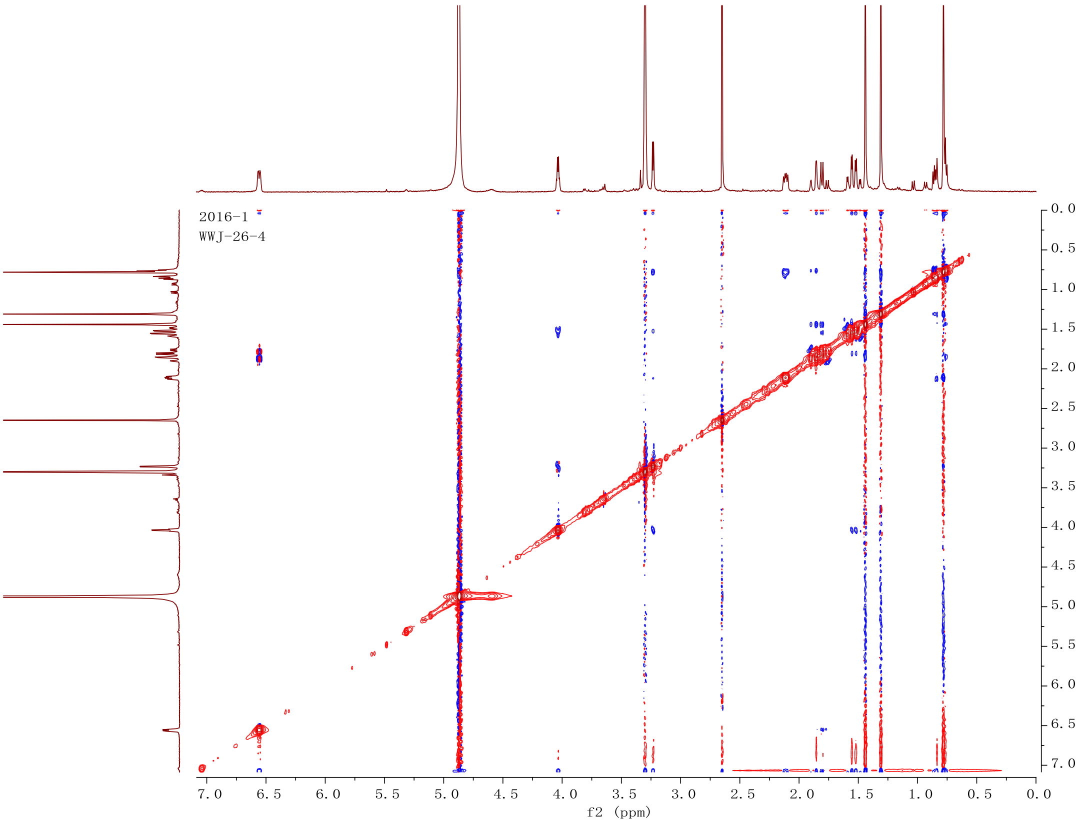

Figure S10. (+)-HR-ESI-MS Spectrum of **2**

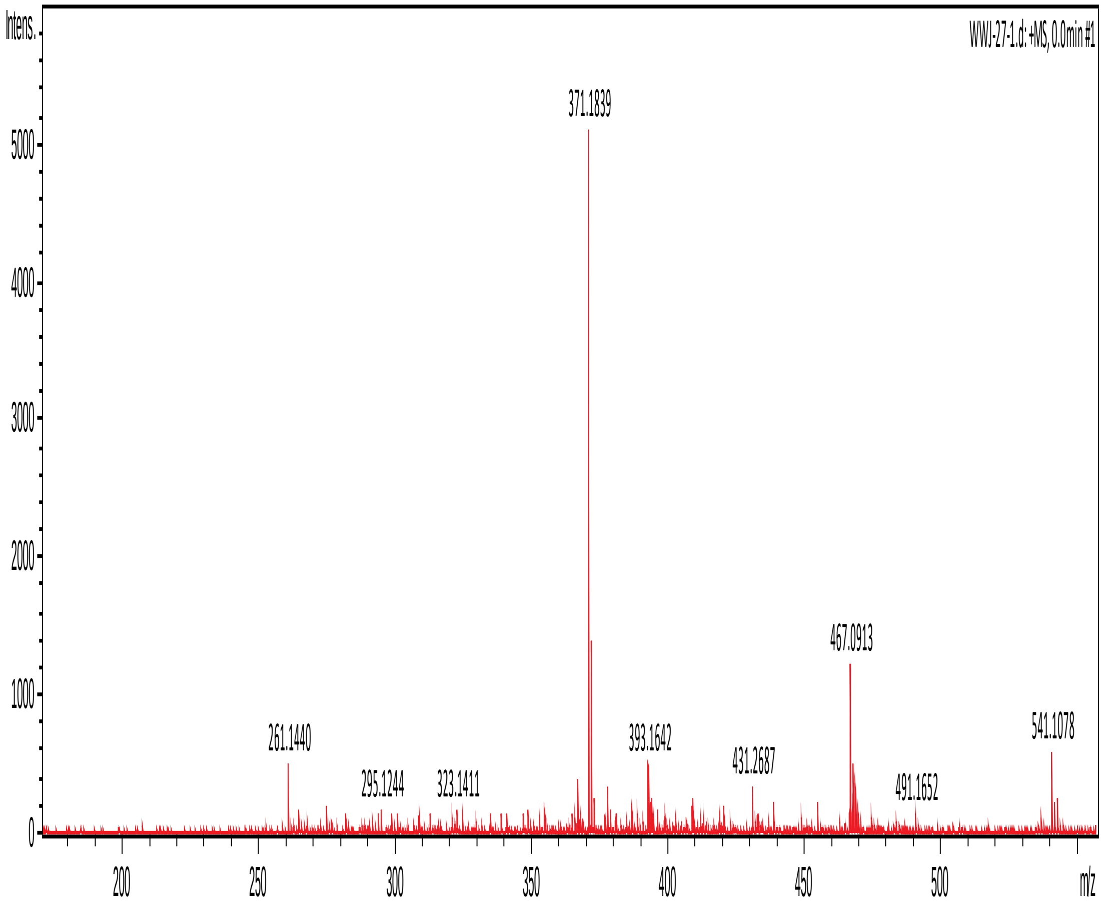

Figure S11. IR Spectrum of **2**

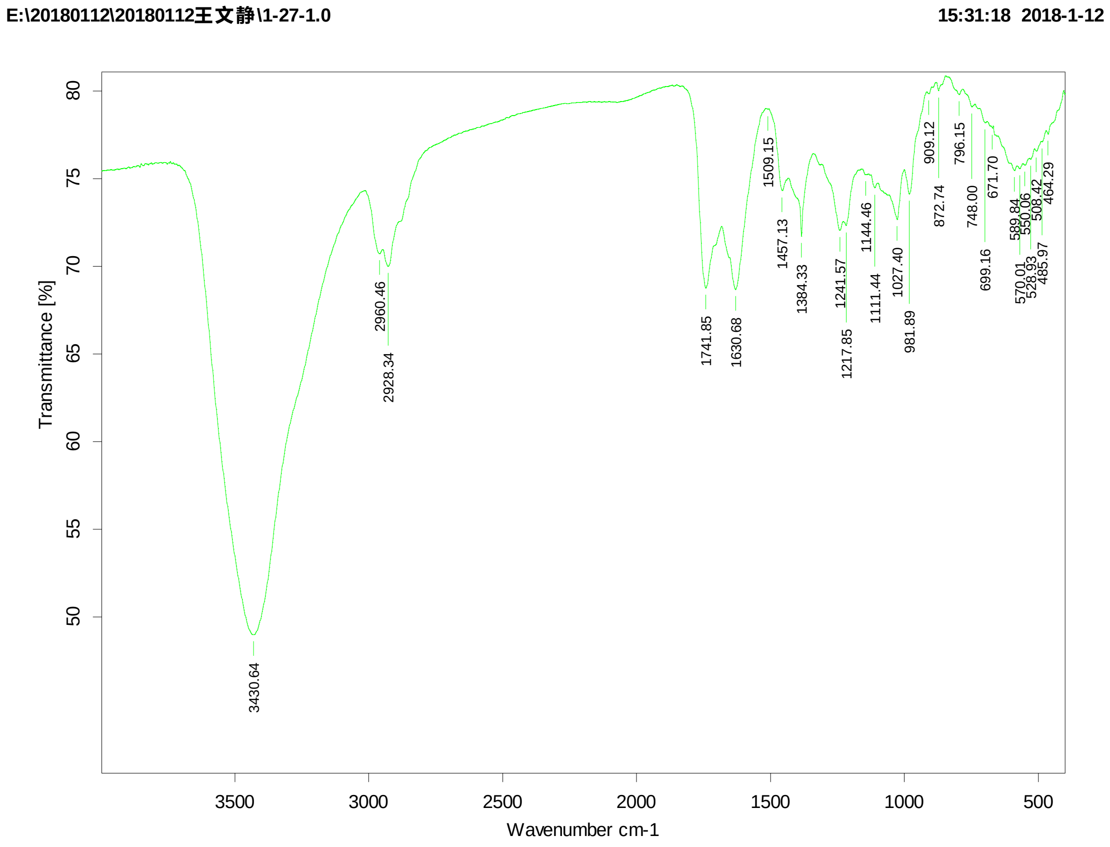

Figure S12. UV Spectrum of **2**

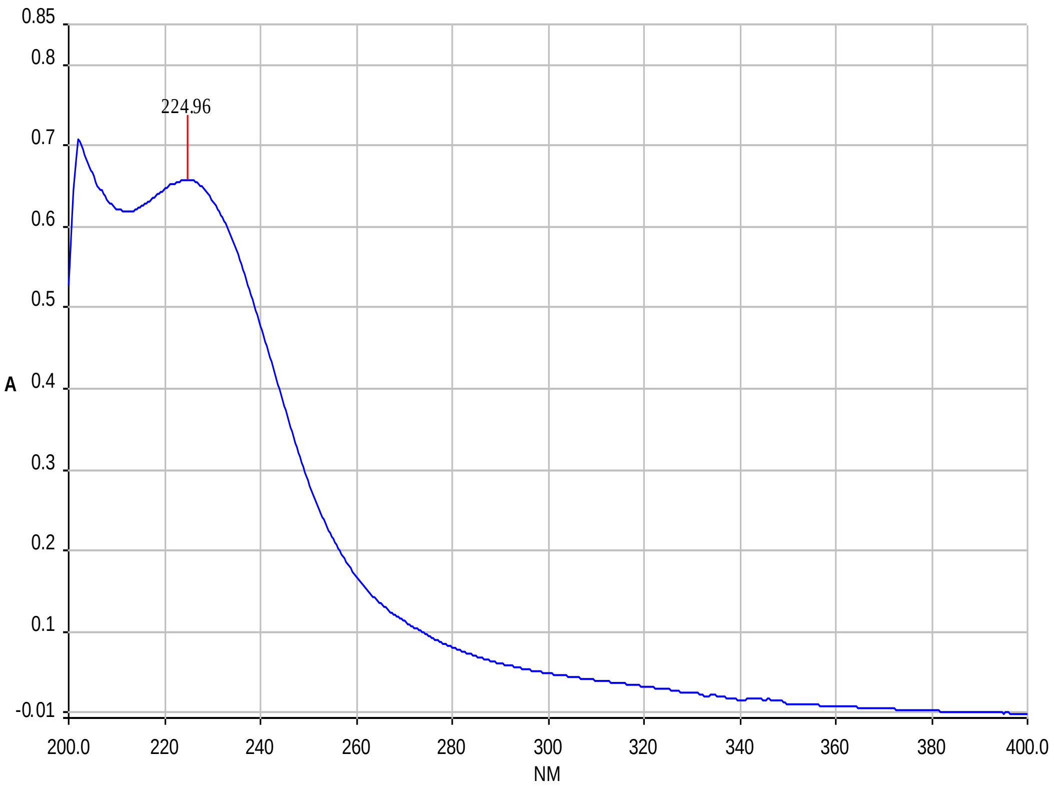

Figure S13. ^{1}H NMR Spectrum of **2** in CD_3OD

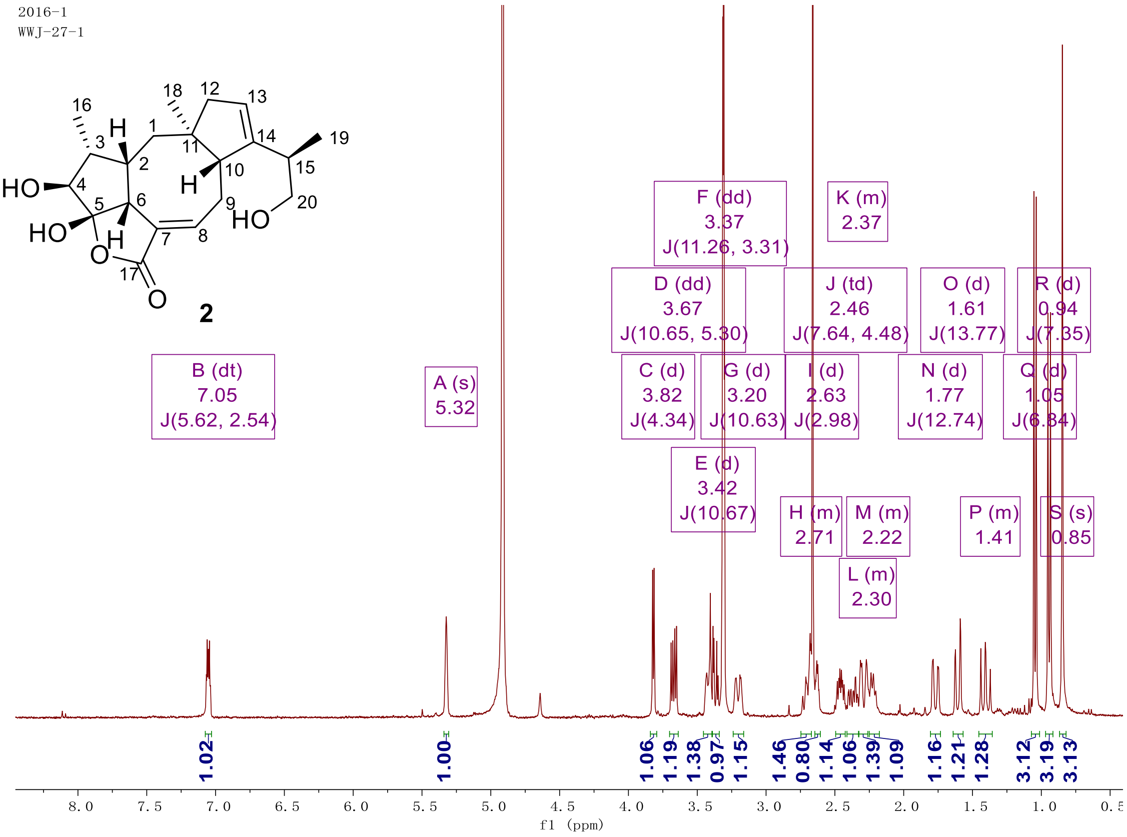

Figure S14. ^{13}C NMR Spectrum of **2** in CD_3OD

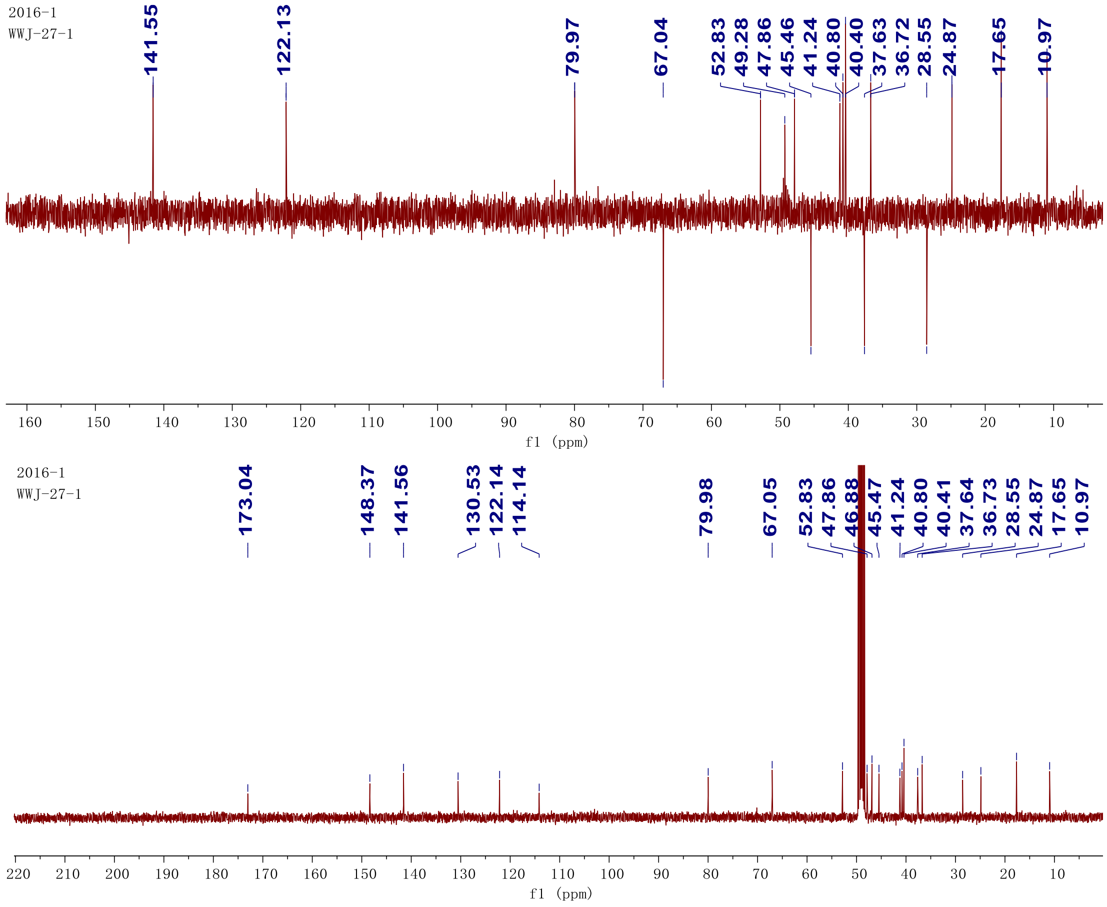

Figure S15. HSQC Spectrum of **2** in CD_3OD

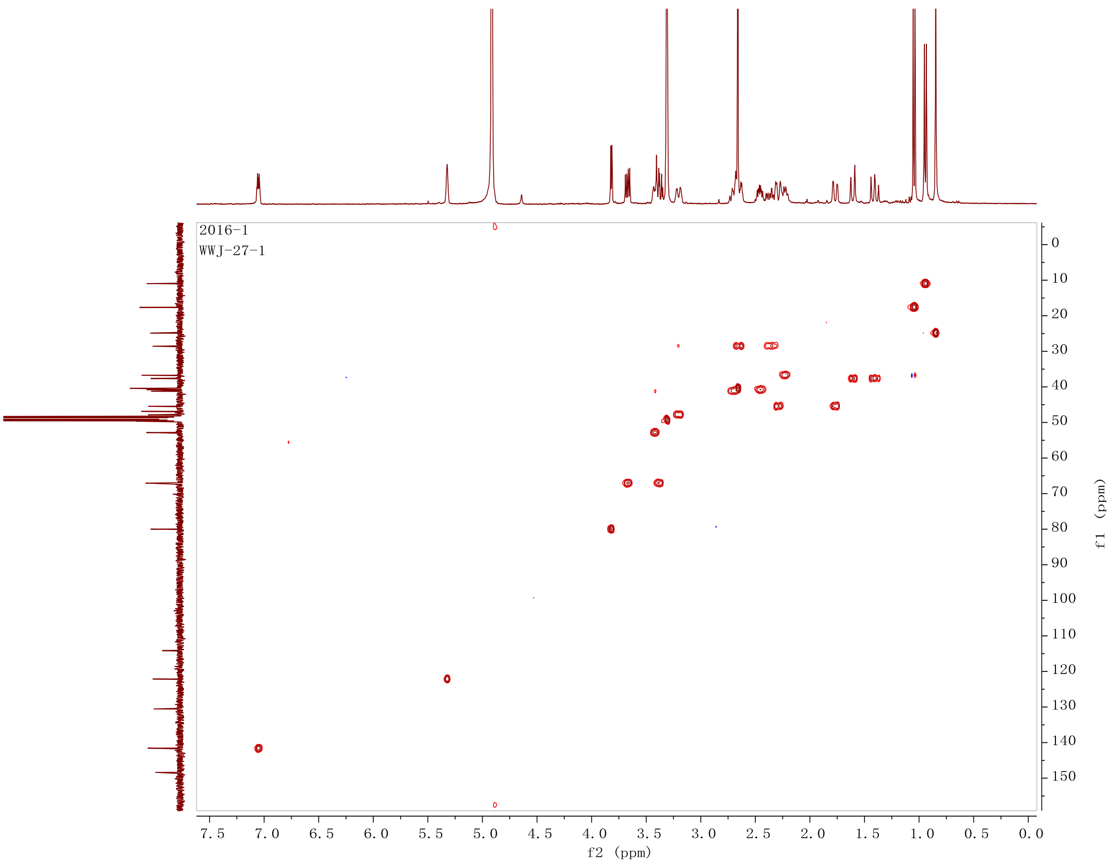

Figure S16. HMBC Spectrum of **2** in CD_3OD

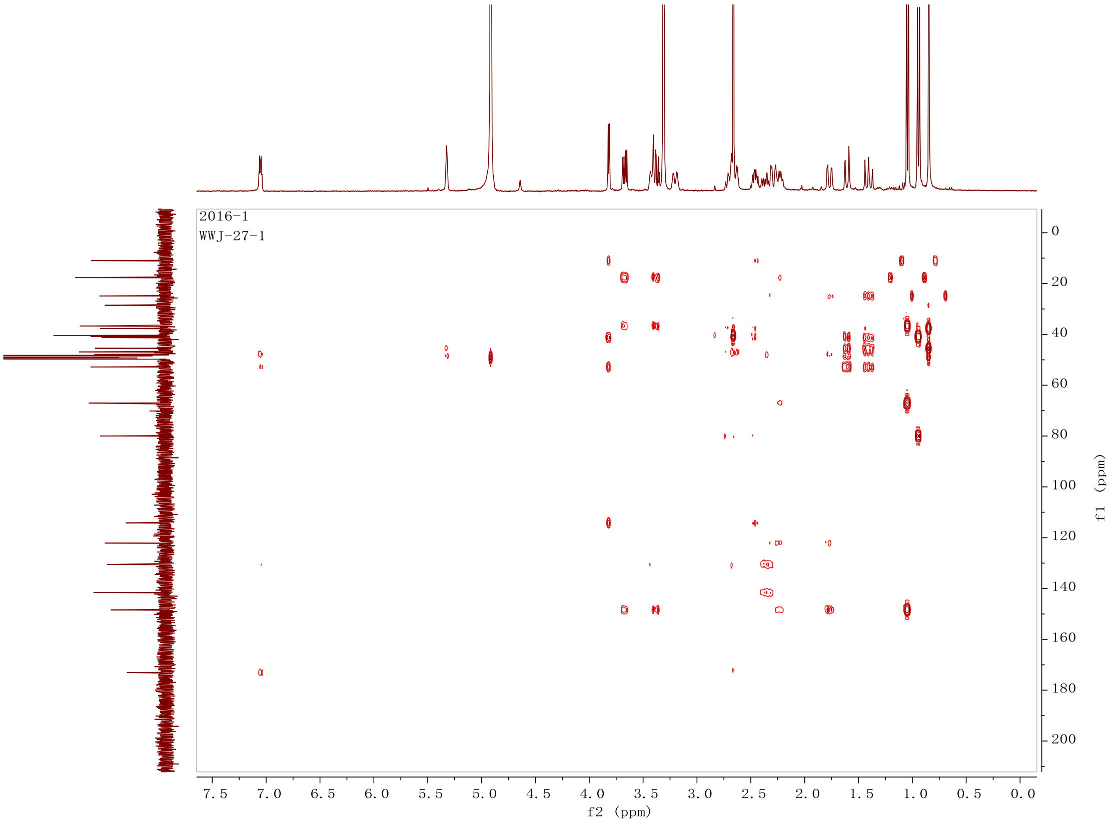

Figure S17. ^1H–^1H COSY Spectrum of **2** in CD_3OD

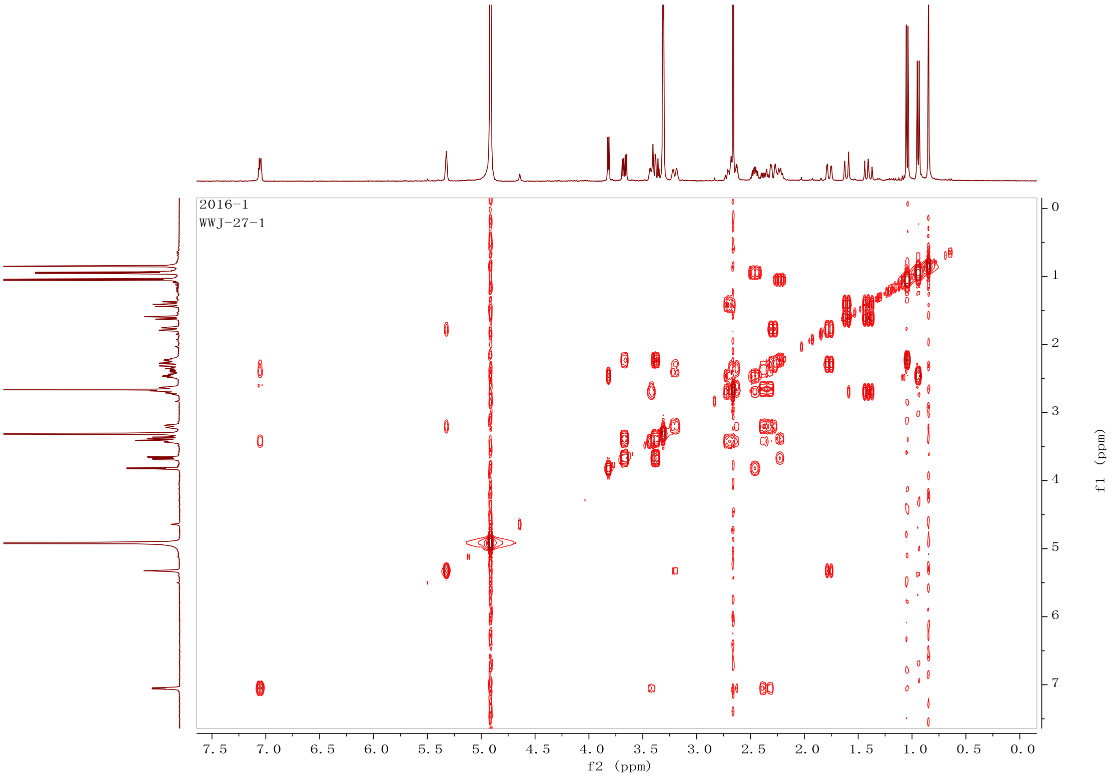

Figure S18. NOESY Spectrum of **2** in CD_3OD

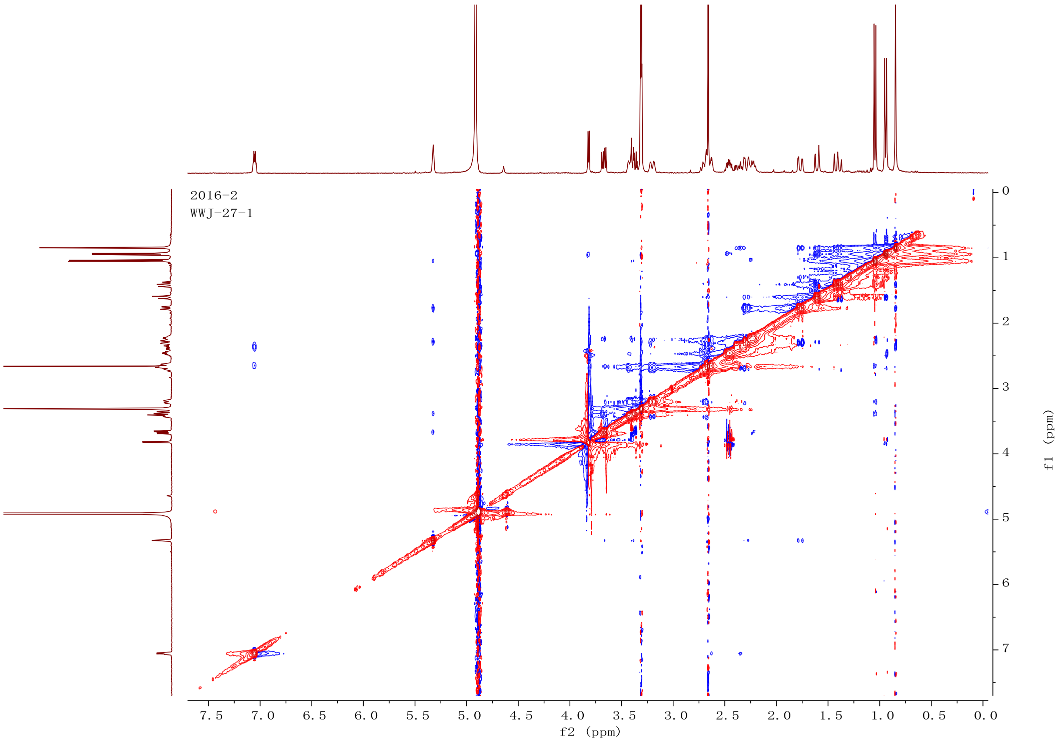

NMR calculations

The conformations of **2** generated by BALLOON[1, 2] were subjected to semiempirical PM3 quantum mechanical geometry optimizations using the Gaussian 09 program[3].

Duplicate conformations were identified and removed when the root-mean-square (RMS) distance was less than 0.5 Å for any two geometry-optimized conformations. The remaining conformations were further optimized at the B3LYP/6-31G(d) level in dimethylsulfoxide with the IEFPCM solvation model using Gaussian 09, and the duplicate conformations emerging after these calculations were removed according to the same RMS criteria above. The harmonic vibrational frequencies were calculated to confirm the stability of the final conformers. The NMR chemical shifts were calculated for each conformer at the B3LYP/6-311++G(d,p)//B3LYP/6-31G(d) level with dimethylsulfoxide as solvent by the IEFPCM solvation model implemented in Gaussian 09 program, which were then combined using Boltzmann weighting according to their population contributions.

Figure S19. Optimized geometries of predominant conformers of **2a** at the B3LYP/6-31G(d,p) level.

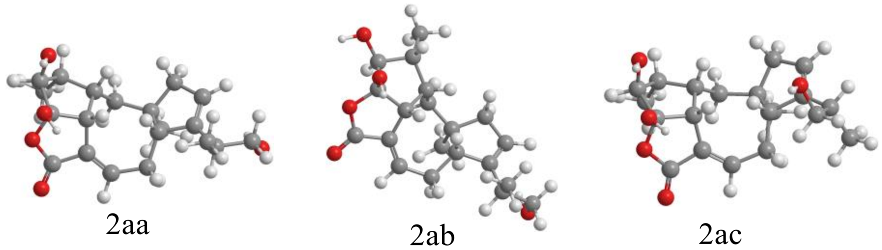

2aa 2ab 2ac

Table S1. Conformational distribution of **2a**

**Species**	**2aa**	**2ab**	**2ac**
Distribution (%)	82.07	16.40	1.53

[1] M.J.V. And, M.S. Johnson, Generating Conformer Ensembles Using a Multiobjective Genetic Algorithm, Journal of Chemical Information & Modeling, 47 (2007) 2462-2474.

[2] J.S. Puranen, M.J. Vainio, M.S. Johnson, Accurate conformation-dependent molecular electrostatic potentials for high-throughput in silico drug discovery, Journal of Computational Chemistry, 31 (2010) 1722-1732.

[3] G.W.S. Frisch MJT, H. B.; Scuseria, G. E.; Robb, M. A.; Cheeseman, J. R.; Scalmani, G.; Barone, V.; Mennucci, B.; Petersson, G. A.; Nakatsuji, H.; Caricato, M.; Li, X.; Hratchian, H. P.; Izmaylov, A. F.; Bloino, J.; Zheng, G.; Sonnenberg, J. L.; Hada, M.; Ehara, M.; Toyota, K.; Fukuda, R.; Hasegawa, J.; Ishida, M.; Nakajima, T.; Honda, Y.; Kitao, O.; Nakai, H.; Vreven, T.; Montgomery, J. A., Jr.; Peralta, J. E.; Ogliaro, F.; Bearpark, M.; Heyd, J. J.; Brothers, E.; Kudin, K. N.; Staroverov, V. N.; Kobayashi, R.; Normand, J.; Raghavachari, K.; Rendell, A.; Burant, J. C.; Iyengar, S. S.; Tomasi, J.; Cossi, M.; Rega, N.; Millam, N. J.; Klene, M.; Knox, J. E.; Cross, J. B.; Bakken, V.; Adamo, C.; Jaramillo, J.; Gomperts, R.; Stratmann, R. E.; Yazyev, O.; Austin, A. J.; Cammi, R.; Pomelli, C.; Ochterski, J. W.; Martin, R. L.; Morokuma, K.; Zakrzewski, V. G.; Voth, G. A.; Salvador, P.; Dannenberg, J. J.; Dapprich, S.; Daniels, A. D.; Farkas, Ö.; Foresman, J. B.; Ortiz, J. V.; Cioslowski, J.; Fox, D. J., (2009) Gaussian 09, Revision D 01, Gaussian, Inc, Wallingford, CT.

Table S2. Compound **2a** structure optimized at B3LYP/6-31G*

Conformation 2aa							
Atom	X	Y	Z	Atom	X	Y	Z
C	0.176	1.588	-1.42	H	-1.67	2.214	-0.63
C	-3.135	-1.405	0.622	H	-1.821	1.186	-2.024
C	1.288	1.228	-0.756	H	-1.333	-2.442	1.192
C	-2.951	-0.102	0.352	H	-2.158	-3.203	-0.167
C	2.624	1.638	-1.255	H	0.782	-1.25	-1.701
C	-1.263	1.298	-1.086	H	0.486	-2.723	-0.812
C	-1.918	-2.231	0.283	H	0.895	0.802	1.328
C	0.391	-1.629	-0.751	H	-0.93	0.285	0.76
C	1.492	0.418	0.498	H	0.908	-1.475	1.341
C	-1.516	0.106	-0.15	H	2.792	-2.699	0.792
C	1.312	-1.145	0.377	H	4.672	-0.759	0.848
C	2.765	-1.728	0.283	H	3.315	-1.026	-1.74
C	3.605	-0.745	1.113	H	2.757	-2.701	-1.673
C	-1.126	-1.313	-0.693	H	4.364	-2.279	-1.074
C	2.995	0.641	0.823	H	-1.211	-0.866	-2.846
C	3.324	-1.937	-1.134	H	-2.764	-1.38	-2.161
C	-1.682	-1.536	-2.118	H	-1.476	-2.563	-2.439
C	-3.514	1.812	1.898	H	-3.594	1.166	2.78
C	-5.368	0.579	0.702	H	-2.484	2.18	1.846
C	-3.906	1.042	0.62	H	-4.171	2.678	2.045
O	2.923	2.244	-2.261	H	-5.503	-0.09	1.567
O	3.583	1.198	-0.38	H	-6.014	1.449	0.862
O	3.418	-1.063	2.487	H	-3.849	1.742	-0.225
O	3.285	1.484	1.898	H	3.58	-0.24	2.982
O	-5.829	-0.034	-0.499	H	2.707	2.265	1.873
H	0.336	2.199	-2.308	H	-5.202	-0.749	-0.699
H	-4.023	-1.835	1.079				

Conformation **2ab**							
**Atom**	**X**	**Y**	**Z**	**Atom**	**X**	**Y**	**Z**
C	0.045	2.018	-0.855	H	-1.802	2.32	0.098
C	-3.154	-1.549	0.115	H	-1.945	1.733	-1.534
C	1.195	1.545	-0.345	H	-1.362	-2.708	0.396
C	-3	-0.228	0.298	H	-2.094	-2.959	-1.185
C	2.461	2.228	-0.734	H	0.939	-0.693	-1.748
C	-1.373	1.591	-0.609	H	0.574	-2.332	-1.267
C	-1.902	-2.198	-0.418	H	1.153	0.741	1.643
C	0.451	-1.286	-0.966	H	-1.002	0.125	0.884

C	1.534	0.459	0.655	H	0.493	-1.396	1.174
C	-1.563	0.174	-0.059	H	2.674	-1.917	1.649
C	1.163	-1.056	0.378	H	3.513	-0.703	-1.003
C	2.519	-1.813	0.566	H	2.628	-3.161	-1.156
C	3.574	-0.815	0.091	H	1.843	-3.866	0.27
C	-1.077	-1.001	-0.981	H	3.601	-3.653	0.235
C	3.093	0.511	0.71	H	-0.999	0.094	-2.886
C	2.649	-3.201	-0.061	H	-2.581	-0.634	-2.541
C	-1.499	-0.777	-2.448	H	-1.223	-1.651	-3.05
C	-3.663	1.053	2.368	H	-3.746	0.15	2.982
C	-5.447	0.247	0.768	H	-2.643	1.437	2.473
C	-3.999	0.742	0.894	H	-4.348	1.808	2.771
O	2.621	3.168	-1.485	H	-5.58	-0.678	1.352
O	3.515	1.642	-0.103	H	-6.124	0.997	1.191
O	4.869	-1.227	0.479	H	-3.944	1.684	0.331
O	3.641	0.644	1.979	H	5.502	-0.562	0.164
O	-5.86	0.064	-0.584	H	3.215	1.398	2.419
H	0.161	2.867	-1.528	H	-5.198	-0.516	-0.996
H	-4.042	-2.122	0.368				

Conformation **2ac**							
**Atom**	**X**	**Y**	**Z**	**Atom**	**X**	**Y**	**Z**
C	-0.048	-0.906	1.898	H	-1.968	-1.581	1.398
C	-3.155	1.569	-0.991	H	-1.971	-0.143	2.372
C	1.061	-0.883	1.14	H	-1.288	2.234	-1.833
C	-3.089	0.404	-0.333	H	-2.02	3.436	-0.772
C	2.378	-1.24	1.725	H	0.784	1.783	1.283
C	-1.472	-0.606	1.513	H	0.585	2.941	-0.009
C	-1.872	2.356	-0.906	H	0.624	-1.087	-0.97
C	0.397	1.893	0.264	H	-1.128	-0.284	-0.564
C	1.283	-0.524	-0.306	H	0.823	1.066	-1.683
C	-1.67	0.232	0.237	H	2.819	2.223	-1.562
C	1.235	1.01	-0.669	H	4.532	0.2	-1.035
C	2.733	1.462	-0.777	H	3.308	1.371	1.358
C	3.459	0.201	-1.272	H	2.876	2.984	0.781
C	-1.143	1.711	0.307	H	4.422	2.265	0.321
C	2.747	-0.97	-0.564	H	-1.213	1.975	2.493
C	3.363	2.046	0.499	H	-2.732	2.382	1.67
C	-1.641	2.418	1.587	H	-1.339	3.473	1.569
C	-4.876	-0.808	1.045	H	-4.196	-1.234	1.79
C	-3.765	-1.985	-0.893	H	-5.231	0.155	1.428
C	-4.201	-0.624	-0.33	H	-5.739	-1.478	0.961
O	2.665	-1.528	2.867	H	-3.028	-2.462	-0.23

O	3.334	-1.184	0.746	H	-4.643	-2.648	-0.93
O	3.248	0.099	-2.676	H	-4.97	-0.244	-1.016
O	2.925	-2.124	-1.33	H	3.319	-0.848	-2.893
O	-3.219	-1.792	-2.198	H	2.271	-2.796	-1.074
H	0.098	-1.221	2.932	H	-2.959	-2.664	-2.534
H	-4.012	1.895	-1.577				

Figure S20. Optimized geometries of predominant conformers of **2b** at the B3LYP/6-31G(d,p) level

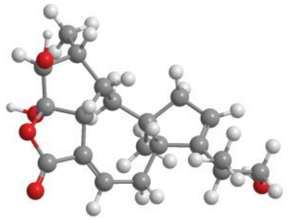

2b

Table S3. Conformational distribution of **2b**

**Species**	**2b**
Distribution (%)	100

Table S4. Compound **2b** structure optimized at B3LYP/6-31G*

Conformation **2b**							
**Atom**	**X**	**Y**	**Z**	**Atom**	**X**	**Y**	**Z**
C	0.098	2.067	-0.819	H	-1.678	1.483	-1.832
C	-3.136	-1.511	0.275	H	-1.908	2.257	-0.299
C	1.233	1.598	-0.286	H	-1.375	-2.671	0.7
C	-2.95	-0.182	0.291	H	-2.114	-3.096	-0.84
C	2.497	2.413	-0.372	H	0.55	-2.586	-0.988
C	-1.292	1.498	-0.804	H	0.979	-1.039	-1.678
C	-1.903	-2.251	-0.171	H	1.778	0.666	1.514
C	0.47	-1.504	-0.829	H	-0.944	0.196	0.837
C	1.671	0.366	0.462	H	0.502	-1.254	1.301
C	-1.5	0.144	-0.11	H	2.44	-2.184	1.904
C	1.192	-1.1	0.466	H	4.64	-1.219	0.436
C	2.547	-1.864	0.861	H	2.088	-3.855	0.094
C	3.717	-0.8	0.863	H	3.03	-2.855	-1.026
C	-1.045	-1.157	-0.876	H	3.804	-3.569	0.4
C	3.111	0.274	-0.029	H	-2.539	-0.917	-2.471
C	2.883	-3.105	0.029	H	-0.924	-0.323	-2.907
C	-1.465	-1.103	-2.36	H	-1.237	-2.059	-2.846

C	-3.563	1.377	2.178	H	-2.545	1.779	2.204
C	-5.385	0.37	0.743	H	-3.62	0.564	2.911
C	-3.933	0.867	0.769	H	-4.246	2.174	2.494
O	2.643	3.573	-0.68	H	-5.509	-0.471	1.445
O	3.581	1.632	0.005	H	-6.048	1.174	1.081
O	3.97	-0.243	2.145	H	-3.893	1.723	0.081
O	3.144	-0.164	-1.368	H	4.205	-0.973	2.741
O	-5.833	0.018	-0.564	H	4.078	-0.254	-1.627
H	0.179	3.037	-1.311	H	-5.181	-0.611	-0.916
H	-4.037	-2.025	0.598				

Table S5. DFT Calculation Result for C shifts of **2a**

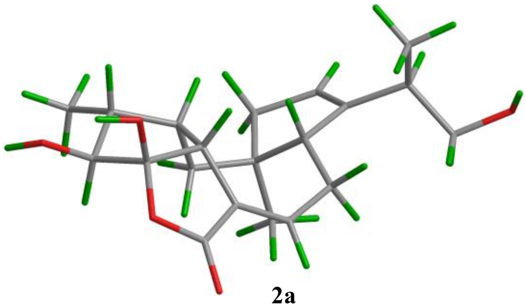

2a

Position	Expt	Calc	Corrected	Δ δ
1	37.6	39.1885	35.91871	-1.68129
2	41.2	45.6765	42.12915	0.929147
3	40.8	49.6043	45.88891	5.088914
4	80	85.9934	80.72128	0.721275
5	114.1	118.368	111.7109	-2.38912
6	52.8	57.9146	53.8437	1.043696
7	130.5	133.529	126.2233	-4.27671
8	141.6	156.397	148.113	6.512984
9	28.6	31.411	28.47393	-0.12607
10	47.9	51.898	48.08449	0.184489
11	46.9	53.1627	49.29508	2.395084
12	45.5	46.5605	42.97533	-2.52467
13	122.1	128.429	121.3415	-0.75853
14	148.4	159.336	150.9263	2.526253
15	36.7	41.5995	38.22656	1.526562
16	11	15.0823	12.84378	1.843778
17	173	178.622	169.3872	-3.61281
18	24.9	25.0123	22.34897	-2.55103
19	17.6	17.6939	15.34365	-2.25635

20	67	68.9473	64.40441	-2.59559
			Average	2.28
			Max	6.4

Figure S21. The ^{13}C NMR correlation of experimental data and calculated dada of **2a**

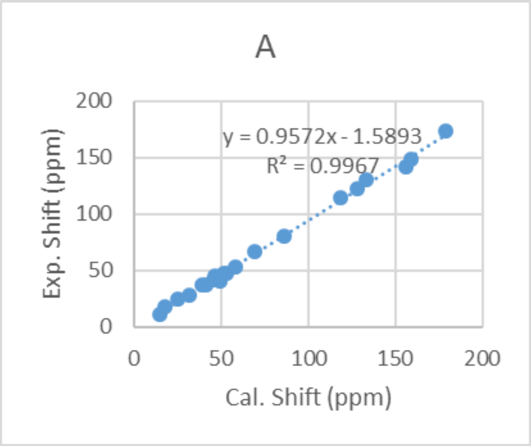

Table S6. DFT Calculation Result for C shifts of **2b**

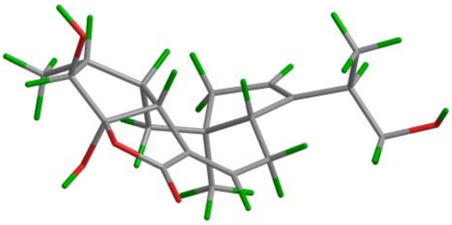

2b

Position	Expt	Calc	Corrected	Δ δ
1	37.6	36.2084	33.66415	-3.93585
2	41.2	38.6217	35.96614	-5.23386
3	40.8	58.7194	55.13687	14.33687
4	80	85.4123	80.59862	0.598618
5	114.1	115.16	108.9743	-5.12574
6	52.8	55.4376	52.00644	-0.79356
7	130.5	133.724	126.682	-3.81796
8	141.6	148.588	140.8605	-0.73954
9	28.6	31.0093	28.70485	0.104848
10	47.9	49.9625	46.78387	-1.11613
11	46.9	51.5738	48.32085	1.420851
12	45.5	48.1855	45.08883	-0.41117
13	122.1	127.793	121.0246	-1.07541
14	148.4	159.298	151.0765	2.676486
15	36.7	40.8487	38.09043	1.390425

16	11	17.018	15.35887	4.358869
17	173	184.931	175.5272	2.527215
18	24.9	24.991	22.96413	-1.93587
19	17.6	17.5293	15.84659	-1.75341
20	67	69.6091	65.52431	-1.47569
			Average	2.74
			Max	14.7

Figure S22. The ^{13}C NMR correlation of experimental data and calculated dada of **2b**

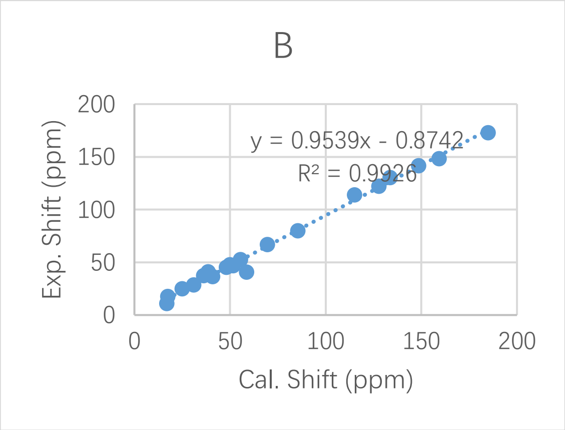

ECD calculations

Table S7. Details for ECD calculation of **2**

Important thermodynamic parameters (a.u.) and Boltzmann distributions of the optimized **2a**

Conformations	⊿G	%
**2a1**	0.40426	66.18%
**2a2**	0.406720	16.53%
**2a3**	0.406721	14.86%
**2a4**	0.406448	2.43%

Important thermodynamic parameters (a.u.) and Boltzmann distributions of the optimized **2b**

Conformations	⊿G	%
**2b1**	0.407348	16.06%
**2b2**	0.406720	79.48%
**2b3**	0.405692	3.88%

Optimized Z-matrixes of compound **2a**

Conformation **2a1**							
Atom	X	Y	Z	Atom	X	Y	Z
C	-0.035	-2.063	-0.754	H	1.875	-1.755	-1.61
C	3.033	1.718	-0.343	H	1.895	-2.196	0.074
C	-1.172	-1.592	-0.215	H	1.208	2.827	-0.066

C	2.959	0.418	-0.023	H	1.826	2.937	-1.71
C	-2.433	-2.336	-0.493	H	-0.811	2.2	-1.52
C	1.374	-1.556	-0.655	H	-1.131	0.509	-1.82
C	1.721	2.255	-0.856	H	-1.067	-0.618	1.695
C	-0.617	1.193	-1.135	H	1.029	0.018	0.726
C	-1.503	-0.436	0.708	H	-0.492	1.486	0.981
C	1.528	-0.086	-0.248	H	-2.61	1.987	1.607
C	-1.211	1.058	0.276	H	-3.794	0.594	-0.823
C	-2.57	1.795	0.523	H	-2.861	2.992	-1.285
C	-3.647	0.74	0.253	H	-3.735	3.582	0.132
C	0.92	0.972	-1.238	H	-1.99	3.833	0.012
C	-3.044	-0.544	0.86	H	0.896	1.427	-3.363
C	-2.797	3.123	-0.199	H	2.33	0.518	-2.858
C	1.251	0.626	-2.704	H	0.763	-0.301	-3.025
C	4.908	-1.139	-0.486	H	5.688	-1.741	-0.009
C	4.938	0.378	1.547	H	5.392	-0.398	-1.133
C	4.051	-0.435	0.585	H	4.305	-1.796	-1.12
O	-2.593	-3.344	-1.153	H	4.305	0.91	2.273
O	-3.479	-1.71	0.105	H	5.509	1.13	0.992
O	-4.928	1.017	0.82	H	3.57	-1.217	1.194
O	-3.423	-0.692	2.184	H	-4.836	1.757	1.442
O	5.917	-0.418	2.213	H	-4.348	-0.372	2.224
H	-0.158	-2.971	-1.345	H	5.441	-1.049	2.777
H	3.908	2.349	-0.212				

Conformation **2a2**							
Atom	X	Y	Z	Atom	X	Y	Z
C	0.045	-0.923	1.919	H	-1.847	-1.665	1.386
C	-3.229	1.488	-0.822	H	-1.911	-0.268	2.432
C	1.134	-0.86	1.134	H	-1.414	2.196	-1.754
C	-3.087	0.317	-0.183	H	-2.126	3.379	-0.657
C	2.477	-1.172	1.68	H	0.81	1.814	1.267
C	-1.394	-0.677	1.555	H	0.527	2.949	-0.029
C	-1.958	2.303	-0.8	H	0.633	-1.089	-0.957
C	0.378	1.9	0.264	H	-1.115	-0.306	-0.517
C	1.3	-0.503	-0.32	H	0.752	1.065	-1.69
C	-1.638	0.186	0.308	H	2.725	2.268	-1.652
C	1.202	1.027	-0.692	H	4.504	0.292	-1.168
C	2.684	1.513	-0.857	H	3.33	1.457	1.257
C	3.422	0.265	-1.366	H	2.848	3.055	0.679
C	-1.154	1.675	0.375	H	4.392	2.361	0.176
C	2.765	-0.914	-0.622	H	-1.14	1.907	2.563

C	3.343	2.124	0.39	H	-2.699	2.293	1.809
C	-1.614	2.355	1.683	H	-1.336	3.417	1.669
C	-5.563	-0.268	-0.218	H	-5.747	0.106	-1.232
C	-3.883	-1.921	-1.062	H	-6.282	-1.072	-0.025
C	-4.122	-0.781	-0.058	H	-5.766	0.548	0.482
O	2.81	-1.442	2.815	H	-4.681	-2.671	-0.947
O	3.401	-1.098	0.67	H	-3.946	-1.519	-2.085
O	3.163	0.142	-2.76	H	-4.037	-1.226	0.944
O	2.947	-2.074	-1.38	H	3.247	-0.806	-2.969
O	-2.606	-2.515	-0.828	H	2.314	-2.755	-1.101
H	0.226	-1.229	2.95	H	-2.49	-3.227	-1.476
H	-4.128	1.819	-1.333				

Conformation **2a3**							
Atom	X	Y	Z	Atom	X	Y	Z
C	-0.235	-1.843	0.872	H	-2.162	-1.293	1.56
C	-2.902	2.147	-0.066	H	-2.107	-1.902	-0.069
C	0.967	-1.538	0.356	H	-0.968	3.054	-0.318
C	-2.951	0.824	-0.275	H	-1.658	3.357	1.272
C	2.135	-2.367	0.772	H	0.896	2.37	1.281
C	-1.585	-1.231	0.63	H	1.037	0.692	1.747
C	-1.576	2.6	0.481	H	1.065	-0.761	-1.643
C	0.625	1.353	0.975	H	-1.032	0.167	-0.871
C	1.454	-0.516	-0.649	H	0.635	1.451	-1.166
C	-1.59	0.2	0.075	H	2.847	1.664	-1.701
C	1.274	1.033	-0.382	H	3.653	0.463	0.977
C	2.708	1.617	-0.611	H	2.255	3.734	-0.38
C	3.647	0.511	-0.121	H	2.996	3.005	1.059
C	-0.929	1.284	1.007	H	3.978	3.37	-0.366
C	2.987	-0.762	-0.683	H	-2.452	1.102	2.579
C	2.997	3.005	-0.036	H	-0.98	0.162	2.9
C	-1.361	1.098	2.477	H	-0.963	1.918	3.086
C	-3.754	-0.645	-2.198	H	-3.088	-1.492	-1.996
C	-4.921	-0.759	0.078	H	-3.251	0.014	-2.913
C	-4.132	0.124	-0.917	H	-4.652	-1.037	-2.689
O	2.163	-3.317	1.527	H	-5.167	-0.164	0.963
O	3.264	-1.905	0.175	H	-4.324	-1.619	0.41
O	4.988	0.573	-0.603	H	-4.836	0.915	-1.212
O	3.443	-1.028	-1.962	H	5.201	1.492	-0.83
O	-6.172	-1.199	-0.455	H	4.361	-0.684	-1.983
H	-0.232	-2.692	1.556	H	-5.988	-1.913	-1.085
H	-3.704	2.837	-0.319				

Conformation **2a4**							
Atom	X	Y	Z	Atom	X	Y	Z
C	0.082	-1.506	-1.294	H	1.954	-1.853	-0.413
C	2.907	2.028	0.614	H	2.035	-0.902	-1.867
C	-1.085	-1.261	-0.675	H	0.971	2.868	1.048
C	2.915	0.698	0.451	H	1.736	3.61	-0.356
C	-2.346	-1.863	-1.176	H	-0.882	1.183	-1.766
C	1.462	-1.021	-0.939	H	-0.798	2.738	-0.976
C	1.61	2.657	0.175	H	-0.802	-0.668	1.385
C	-0.566	1.672	-0.838	H	0.952	0.052	0.829
C	-1.423	-0.415	0.524	H	-1.101	1.585	1.252
C	1.54	0.25	-0.075	H	-3.114	2.519	0.616
C	-1.441	1.147	0.307	H	-4.734	0.361	0.761
C	-2.955	1.533	0.163	H	-3.382	0.655	-1.822
C	-3.679	0.498	1.038	H	-3.03	2.387	-1.846
C	0.978	1.562	-0.733	H	-4.585	1.808	-1.24
C	-2.891	-0.812	0.836	H	1.185	0.974	-2.846
C	-3.511	1.591	-1.27	H	2.634	1.739	-2.167
C	1.54	1.751	-2.16	H	1.21	2.716	-2.563
C	3.626	-1.211	1.984	H	2.869	-1.912	1.615
C	4.785	-0.881	-0.262	H	4.491	-1.795	2.31
C	4.052	-0.196	0.904	H	3.208	-0.696	2.856
O	-2.54	-2.555	-2.152	H	4.166	-1.671	-0.709
O	-3.375	-1.503	-0.345	H	4.994	-0.137	-1.046
O	-3.558	0.912	2.394	H	4.801	0.467	1.358
O	-3.094	-1.629	1.95	H	-3.623	0.103	2.932
O	6	-1.43	0.25	H	-2.404	-2.312	1.989
H	0.028	-2.179	-2.15	H	6.424	-1.922	-0.47
H	3.719	2.596	1.063				

Optimized Z-matrixes of compound 2b

**Conformation 2b1**							
**Atom**	**X**	**Y**	**Z**	**Atom**	**X**	**Y**	**Z**
**C**	**-0.199**	**2.177**	**0.056**	**H**	**1.723**	**2.379**	**-0.814**
**C**	**3.168**	**-1.295**	**-0.8**	**H**	**1.726**	**2.139**	**0.909**
**C**	**-1.27**	**1.419**	**0.341**	**H**	**2.114**	**-2.037**	**-2.575**
**C**	**2.997**	**-0.181**	**-0.07**	**H**	**1.38**	**-2.481**	**-1.036**
**C**	**-2.628**	**2.02**	**0.366**	**H**	**-0.56**	**-1.311**	**-2.544**
**C**	**1.252**	**1.786**	**-0.021**	**H**	**-0.875**	**0.351**	**-2.111**
**C**	**1.918**	**-1.673**	**-1.558**	**H**	**-0.753**	**-0.346**	**1.48**
**C**	**-0.436**	**-0.579**	**-1.733**	**H**	**1.018**	**-0.234**	**0.608**

C	-1.411	-0.047	0.662	H	-0.817	-1.962	-0.119
C	1.541	0.287	-0.203	H	-2.755	-2.496	-1.257
C	-1.273	-1.059	-0.541	H	-4.586	-1.224	0.27
C	-2.741	-1.449	-0.931	H	-3.41	0.46	-1.821
C	-3.504	-1.369	0.401	H	-4.437	-0.93	-2.177
C	1.089	-0.355	-1.559	H	-2.882	-0.75	-2.995
C	-2.883	-0.167	1.142	H	1.059	1.473	-2.786
C	-3.397	-0.611	-2.04	H	1.31	-0.018	-3.697
C	1.556	0.497	-2.761	H	2.638	0.663	-2.743
C	4.816	1.571	0.127	H	5.404	1.138	-0.691
C	4.956	-0.544	1.497	H	4.164	2.338	-0.302
C	4.005	0.478	0.851	H	5.506	2.066	0.821
O	-2.978	3.152	0.108	H	5.599	-1	0.727
O	-3.537	1.061	0.728	H	5.615	-0.028	2.204
O	-3.233	-2.56	1.131	H	3.454	0.951	1.676
O	-3.086	-0.343	2.512	H	-3.347	-2.335	2.071
O	4.276	-1.542	2.252	H	-2.488	0.235	3.015
H	-0.406	3.233	-0.123	H	3.648	-1.961	1.639
H	4.077	-1.89	-0.839				

Conformation 2b2							
Atom	X	Y	Z	Atom	X	Y	Z
C	-0.042	-1.622	-1.204	H	-1.857	-2.088	-0.262
C	-3.045	1.73	0.76	H	-2.049	-1.159	-1.717
C	1.127	-1.272	-0.642	H	-2.066	3.368	-0.32
C	-2.944	0.396	0.647	H	-1.178	2.748	1.07
C	2.404	-1.787	-1.194	H	0.705	1.138	-1.76
C	-1.441	-1.229	-0.808	H	0.521	2.693	-0.99
C	-1.832	2.45	0.234	H	0.915	-0.676	1.424
C	0.381	1.615	-0.83	H	-0.922	-0.089	0.917
C	1.454	-0.374	0.524	H	0.977	1.598	1.244
C	-1.572	0.045	0.045	H	2.889	2.69	0.56
C	1.33	1.18	0.294	H	4.696	0.686	0.638
C	2.801	1.697	0.104	H	4.354	2.122	-1.347
C	3.64	0.72	0.944	H	2.736	2.558	-1.903
C	-1.145	1.383	-0.667	H	3.244	0.865	-1.895
C	2.957	-0.649	0.751	H	-1.364	0.728	-2.759
C	3.304	1.808	-1.345	H	-2.854	1.385	-2.057
C	-1.764	1.488	-2.079	H	-1.53	2.467	-2.512
C	-3.413	-1.642	2.129	H	-4.227	-2.235	2.561
C	-4.815	-1.201	0.043	H	-2.733	-2.335	1.624
C	-3.983	-0.572	1.18	H	-2.863	-1.175	2.952
O	2.61	-2.5	-2.15	H	-4.183	-1.83	-0.601

**O**	**3.445**	**-1.288**	**-0.444**	**H**	**-5.588**	**-1.849**	**0.473**
**O**	**3.518**	**1.095**	**2.312**	**H**	**-4.695**	**0.032**	**1.759**
**O**	**3.146**	**-1.524**	**1.837**	**H**	**3.588**	**0.271**	**2.828**
**O**	**-5.502**	**-0.217**	**-0.726**	**H**	**4.055**	**-1.872**	**1.784**
**H**	**0.028**	**-2.311**	**-2.045**	**H**	**-4.869**	**0.508**	**-0.871**
**H**	**-3.872**	**2.241**	**1.248**				

**Conformation 2b3**							
**Atom**	**X**	**Y**	**Z**	**Atom**	**X**	**Y**	**Z**
**C**	**0.092**	**1.788**	**-1.247**	**H**	**2.036**	**1.358**	**-1.976**
**C**	**3.231**	**-1.741**	**-0.012**	**H**	**1.986**	**2.133**	**-0.411**
**C**	**-1.041**	**1.438**	**-0.614**	**H**	**2.037**	**-3.264**	**-1.042**
**C**	**3.131**	**-0.405**	**0.014**	**H**	**1.425**	**-2.784**	**0.537**
**C**	**-2.306**	**2.107**	**-1.027**	**H**	**-0.607**	**-2.548**	**-1.022**
**C**	**1.507**	**1.348**	**-1.015**	**H**	**-0.952**	**-0.977**	**-1.703**
**C**	**1.925**	**-2.404**	**-0.369**	**H**	**-0.904**	**0.914**	**1.462**
**C**	**-0.431**	**-1.476**	**-0.878**	**H**	**1.194**	**0.109**	**0.677**
**C**	**-1.353**	**0.515**	**0.545**	**H**	**-0.333**	**-1.288**	**1.254**
**C**	**1.686**	**0.006**	**-0.297**	**H**	**-2.498**	**-1.648**	**1.93**
**C**	**-1.044**	**-1.037**	**0.462**	**H**	**-3.457**	**-0.792**	**-0.819**
**C**	**-2.407**	**-1.707**	**0.837**	**H**	**-2.651**	**-3.285**	**-0.661**
**C**	**-3.459**	**-0.747**	**0.282**	**H**	**-3.565**	**-3.531**	**0.832**
**C**	**1.102**	**-1.256**	**-1.028**	**H**	**-1.815**	**-3.805**	**0.814**
**C**	**-2.903**	**0.634**	**0.678**	**H**	**0.929**	**-0.412**	**-3.051**
**C**	**-2.616**	**-3.165**	**0.427**	**H**	**1.089**	**-2.171**	**-3**
**C**	**1.43**	**-1.238**	**-2.534**	**H**	**2.508**	**-1.149**	**-2.709**
**C**	**5.635**	**-0.008**	**0.231**	**H**	**5.813**	**-0.831**	**0.934**
**C**	**4.06**	**1.105**	**1.827**	**H**	**5.785**	**-0.394**	**-0.782**
**C**	**4.222**	**0.576**	**0.391**	**H**	**6.394**	**0.76**	**0.42**
**O**	**-2.484**	**2.939**	**-1.893**	**H**	**4.106**	**0.258**	**2.53**
**O**	**-3.339**	**1.653**	**-0.265**	**H**	**4.901**	**1.777**	**2.056**
**O**	**-4.741**	**-1.052**	**0.792**	**H**	**4.147**	**1.451**	**-0.27**
**O**	**-3.371**	**0.965**	**1.943**	**H**	**-5.37**	**-0.413**	**0.42**
**O**	**2.82**	**1.8**	**1.95**	**H**	**-2.894**	**1.756**	**2.247**
**H**	**-0.038**	**2.538**	**-2.027**	**H**	**2.744**	**2.105**	**2.868**
**H**	**4.126**	**-2.308**	**0.229**				
